# Structural insights into the bi-specific cross-over dual variable antibody architecture by cryo-EM

**DOI:** 10.1038/s41598-023-35678-4

**Published:** 2023-05-29

**Authors:** David Fernandez-Martinez, Mark D. Tully, Gordon Leonard, Magali Mathieu, Eaazhisai Kandiah

**Affiliations:** 1 European Synchrotron Radiation Facility, 71 Avenue des Martyrs, 38042 Grenoble, France; 2 Sanofi R&D, Bio Structure and Biophysics, Centre de Recherche Vitry-Sur-Seine, 94403 Vitry-Sur-Seine Cedex, France; 3 Present Address: Pathogenesis of Vascular Infections, Department of Cell Biology and Infection, Institut Pasteur, INSERM, 75015 Paris, France

**Keywords:** Structural biology, Electron microscopy, SAXS, Cancer, Cancer therapy, Cancer immunotherapy

## Abstract

Multi-specific antibodies (msAbs) are being developed as next generation antibody-based therapeutics. Knowledge of the three-dimensional structures, in the full antibody context, of their fragment antigen-binding (Fab) moieties with or without bound antigens is key to elucidating their therapeutic efficiency and stability. However, the flexibility of msAbs, a feature essential for their multi specificity, has hindered efforts in this direction. Cross-Over Dual Variable immunoglobulin (CODV_Ig_) is a promising bispecific antibody format, designed to simultaneously target the interleukins IL4 and IL13. In this work we present the biophysical and structural characterisation of a CODV_Fab_:IL13 complex in the full antibody context, using cryo-electron microscopy at an overall resolution of 4.2 Å. Unlike the 1:2 stoichiometry previously observed for CODV_Ig_:IL4, CODV_Ig_:IL13 shows a 1:1 stoichiometry. As well as providing details of the IL13-CODV binding interface, including the residues involved in the epitope-paratope region, the structure of CODV_Fab_:IL13 also validates the use of labelling antibody as a new strategy for the single particle cryo-EM study of msAbs in complex with one, or more, antigens. This strategy reduced the inherent flexibility of the IL13 binding domain of CODV without inducing either structural changes at the epitope level or steric hindrance between the IL4 and IL13 binding regions of CODV_Ig_. The work presented here thus also contributes to the development of methodology for the structural study of msAbs, a promising platform for cancer immunotherapy.

## Introduction

Multi-specific antibodies (msAbs) are antibodies engineered to simultaneously and selectively bind to two or more different targets^1,2^. These molecules enable a wide array of therapeutic strategies, such as signalling modulation by soluble or membrane ligand targeting, effector cell retargeting, piggybacking and cofactor mimetization^3^ that are not available exploiting conventional monospecific antibodies.

Several bi-specific antibodies (bsAbs) formats have been developed^4^ for multitude of biotherapeutic use including cancer^5,6^. Each format has a distinct architecture, molecular range, ligand affinity and half-life in serum, which, in turn, translates into particular advantages and disadvantages for pharmacokinetics, pharmacodynamics, manufacturing, formulation and other drug-design criteria. Indeed, close to 100 bsAbs are currently in development with two already having been brought to market^7^. The formats developed generally fall into two groups: those containing only the relevant fragment antigen-binding (Fab) domains, and those which include a full-antibody construct. Fab fragment-based formats present a greater degree of malleability and adaptability, but have been shown, in many cases, to have suboptimal stability and/or an increased propensity for aggregation, both of which are disadvantageous in drug manufacturing and administration, for which sustained homogeneity and stability are crucial^8^. Increased efforts are therefore being made in the development of full-antibody bsAbs. These make use of antibody constant fragment (Fc) regions as a means of improving stability throughout the drug manufacturing and administration process, while still keeping parental antibody level affinity maturation. The presence of the Fc region also enables the exploration of additional therapeutic strategies such as those involving opsonization and complement-mediated cell immunity^9,10^.

**Figure 1:**
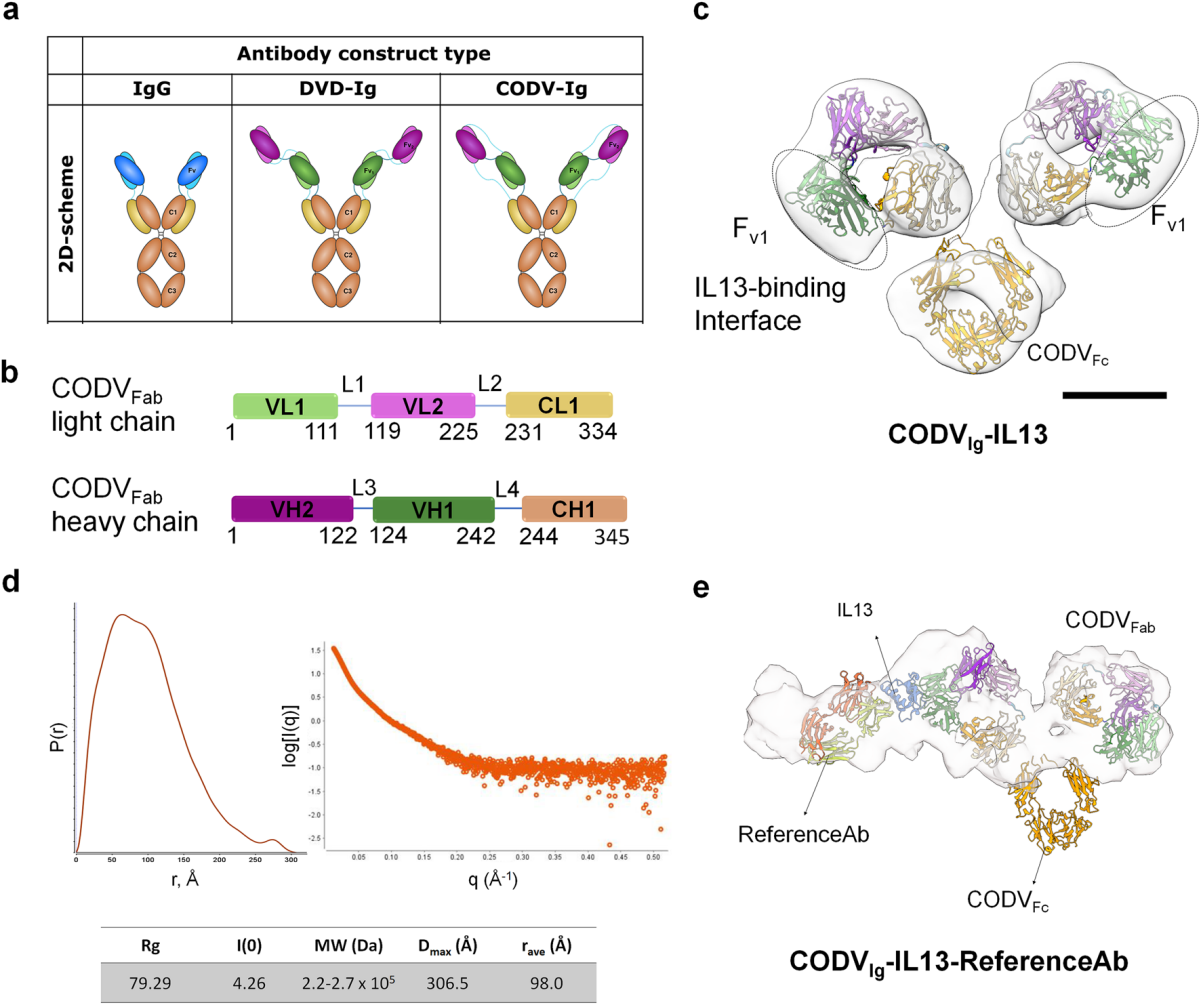
Structure determination of the CODV_Ig_:IL13:RefAb complex (**a**), Schematic representation of mono- and bispecific antibody formats. Heavy chains are in dark colors while light chains are in light colors. Constant domains (CH) are in orange, IL4 binding domains (VH2 and VL2), are in purple and IL13 binding domains (VH1 and VL1) are in green. (**b**), Domain organisation of CODV_Fab_ light and heavy chains. For clarity, the schematics comprises only the domains discerned in the reported structure (**c**), nsEM 3D reconstruction of CODV_Ig_:IL13 obtained show no evidence of antigens bound (scale bar = 50 Å). The reconstructions are fitted with the crystal structures of CODV_Fab_ (PDB: 5HCG)^11^ and IgG-Fc (PDB: 4NQS)^47^. (**d**), SAXS Pair-distance distribution function (left) and scattering curve (right) for CODV_Ig_:IL13:RefAb complex, along with a table showing several calculated values. (**e**), Molecular surface obtained from the aligned, averaged SAXS data fitted with atomic models of CODV_Fab_:IL13:RefAb and IgG-Fc (PDB: 4NQS)^47^. The colour codes in all sub-sections of the figure are similar to that used in (**a**).

The Cross-Over Dual Variable immunoglobulin (CODV_Ig_) was developed as a universal full-antibody bsAbs format in which a second variable domain (Fv) is added to each Fab and both Fvs are connected to each other and to the C1 domain through a series of four modulable-length linkers (Fig. 1a,b)^11^. This architecture confers many advantages, notably the apparent absence of a positional effect, which has been reported for the similar DVD_Ig_ (Dual Variable Domain-Ig) format^12^ (Fig. 1a). CODV_Ig_ should thus allow for antigen binding in any order and in an independent manner, without compromising affinity for either antigen. However, while this desirable phenomenon was observed in Surface Plasmon Resonance assays, other experiments suggest that this is not the case *in vivo*^11^. Moreover, reported IC50 values were orders of magnitude higher when the positions of the Fab domains were swapped^11^. Therefore, it was hypothesized that the orientation of each antibody fragment within the CODV_Ig_ construct is as important as the antibody-ligand interface for a successful therapeutic effect^11^.

To shed light on this hypothesis structural information is required. However, and to our knowledge, all attempts to elucidate the structure of a full antibody CODV_Ig_, either alone or in complex with antigens, by X-ray crystallography have been unsuccessful, presumably due to interdomain flexibility. We therefore adopted a cryo-electron microscopy (cryo-EM) approach, aiming to determine the three-dimensional structures of full-length CODV_Ig_ both in *apo-* and in complex forms with its antigens, interleukins IL13 and IL4, two type 2 cytokines which play important roles in immune response of cells and which have been profoundly linked to cancer. In particular, the atomic structure of CODV_Ig_ in complex with both IL4 and IL13 is of high clinical importance in tumour therapy as a potential JAK/STAT6 pathway targeting system^13,14^.

The main aim of the work described here was to obtain high-resolution details of the various CODV_Ig_ domains, and their relative orientations, in both *apo*- and antigen bound forms. However, preliminary analysis using both negative stain and cryo-electron microscopy (nsEM (Fig. S1), cryo-EM (Fig. S2) suggested that the inherent flexibility of CODV_Ig_ precluded obtaining high-resolution reconstructions. Moreover, no density for antigens was visible in the nsEM and cryo-EM reconstructions obtained. As the biochemical and biophysical characterisation of CODV_Ig_ in complex with IL4 and/or IL13 clearly suggested high affinity bsAbs:antigen binding (Fig. S2), we labelled the CODV_Ig_:IL13 complex with a second antibody which recognises a different IL13 epitope than does CODV_Ig._ This allowed us to obtain a medium resolution (4.2 Å) reconstruction of CODV_Fab_:IL13 in the full antibody context. While the CODV_Ig_ Fc region is still not visible in the reconstruction obtained due to its intrinsic flexibility, our results provide a structural rationale for the unexpected 1:1 stoichiometry for the CODV_Ig_:IL13 complex. Our results also suggest that labelling bsAbs:antigen complexes with partner antibodies may provide a general strategy for the cryo-EM analysis of such flexible moieties in the full antibody context.

## Results

### CODV_Ig_ binding to IL13 exhibits a non-canonical 1:1 stoichiometry

The binding mode and affinity of the interleukins to CODV_Ig_ were characterized a priori to cryo-EM studies by a variety of biophysical methods. For CODV_Ig_ to IL13, results from Size Exclusion Chromatography (SEC) and Mass Photometry (MP) experiments primarily indicate a 1:1 stoichiometry rather than the anticipated 1:2 stoichiometry observed for CODV_Ig_:IL4 (Fig. S3). Although this is puzzling, this appears to agree with the lower CODV_Ig_-binding affinity of IL13 as compared to IL4, as determined using Surface Plasmon Resonance (SPR) (Fig. S4 and Table S1). To shed more light on this observation and to understand the in vitro and in vivo discrepancies of the antigens binding^11^, we instigated the structural study of CODV_Ig_ bound to IL13 using cryo-EM. However, a classical methodology, using ns-EM followed by cryo-EM of *apo*-CODV_Ig_ or CODV_Ig_ bound to either or both interleukins, did not provide reconstructions allowing the elucidation of the structural disposition of the different components. nsEM suffered from a combination of the inherent flexibility of the antibody and the small size of the antigens and uniformly converged to reconstructions with no density for the antigens (Figs. S1 and 1c). Moreover, this flexibility also hindered particle alignment in subsequent cryo-EM refinements (Fig. S2). Nevertheless, our initial EM characterizations allowed us both to characterize the inter-domain flexibility and to develop an image processing workflow that focused solely on the Fab domains.

To obtain structural details of a full antibody-IL3 complex by cryo-EM, we developed a strategy exploiting a partner protein that binds to a different surface of the IL13 antigen than does the CODV_Fab_. This was achieved using the Fab region of the antibody ReferenceAb (RefAb)^15^. Intriguingly, while SEC and MP analyses didn’t provide a strong evidence for 1:2:2 stoichiometry for the CODV_Ig_:IL13:RefAb_Fab_ ternary complex (TC) (Fig. S5a–c), Small Angle X-Ray Scattering (SAXS) experiments coupled with high-resolution size exclusion chromatography (SEC-SAXS) analysis produced an estimated molecular weight of 220–270 kDa and a maximum distance (D_max_) of 307 Å, values consistent with a 1:1:1 stoichiometry (Fig. 1d). This was confirmed by the resulting *ab-initio* molecular envelope which provides an overall view of the shape of the complex and the disposition of its components (Fig. 1e). More quantitative characterisation of the ternary complex by nsEM revealed that the majority of the particles (89%) clearly exhibited a 1:1:1 stoichiometry for the three components (Fig. S5d,e). Additionally, the low-resolution 3D reconstruction obtained from nsEM (Fig. S5f) clearly shows, as does the SAXS envelope, IL13 flanked by densities corresponding to CODV_Fab_ and RefAb_Fab_. These experiments confirmed a reduced flexibility and heterogeneity due to binding of RefAb and prompted a full cryo-EM analysis of the ternary complex (Figs. S5g,h and S6, Table S2).

### Cryo-EM reconstruction of CODV_Fab_:IL13:RefAb_Fab_ deciphers the CODV:IL13 interface

**Figure 2:**
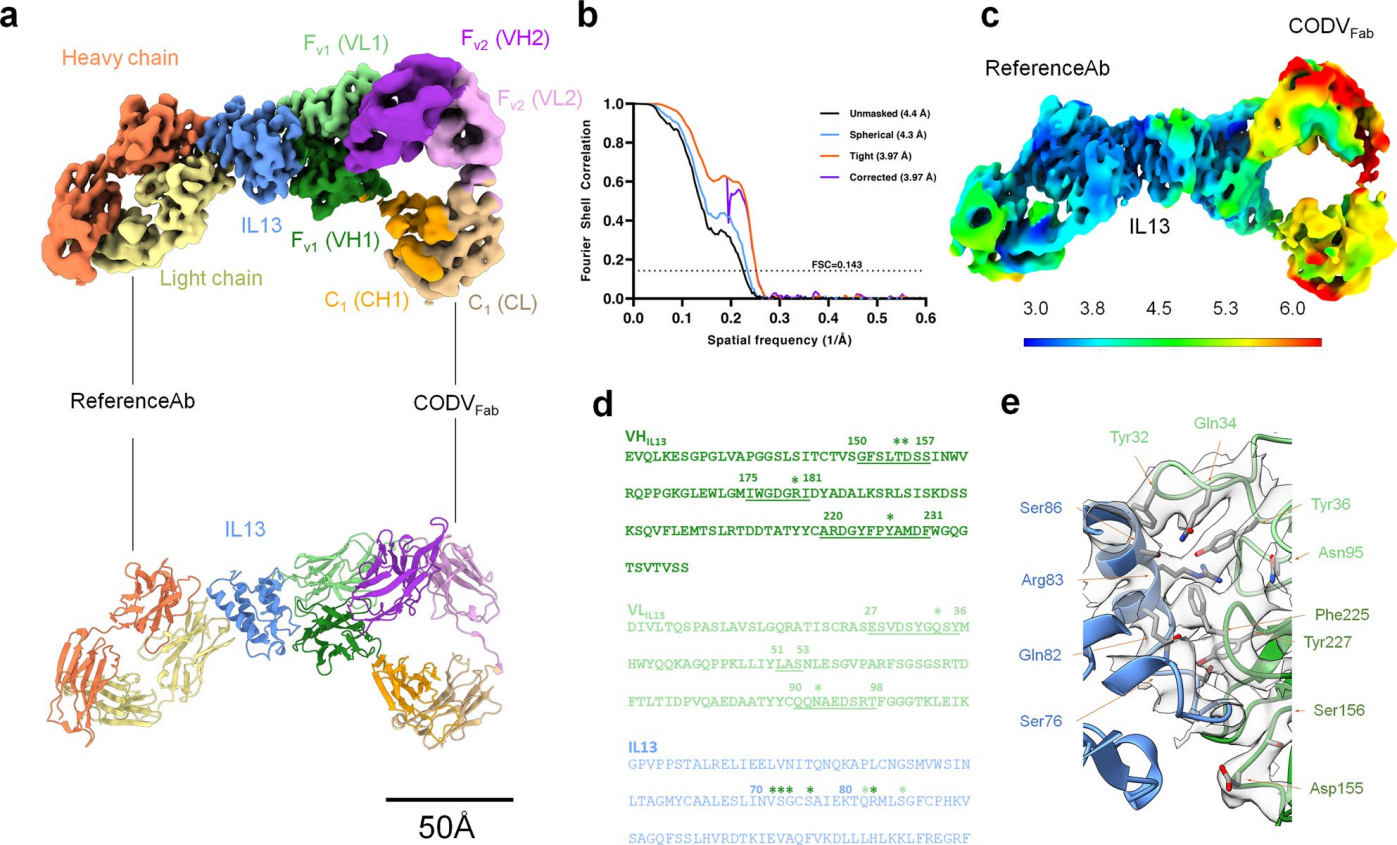
Cryo-EM reconstruction of CODV_Fab_:IL13:RefAb (TC) and its CODV_Fab_:IL13 interface. (**a**) 3D reconstruction of TC with the fitted atomic model shown below. The colour codes of CODV domains are similar to Fig. 1a. The EM density was better-resolved for IL13 (blue) and the antigen–antibody binding interfaces and allowed for more precise modelling of the epitope-paratope residues. RefAb is in orange (heavy chain) and yellow (light chain) (**b**) Global resolutions estimated by the GS-FSC with a 0.143 cutoff. (**c**) Local resolution map of TC as estimated by ResMap. The lower resolution observed is closely related to the intra-flexibility of the Fab domains (**d**) Primary sequences of VH1 and VL1 domains of CODV are shown in green and that of IL13 in blue. CDR loops are underlined and the amino acids involved in hydrogen bonds are marked with asterisks. (**e**), Residues of CODVFab (green) and IL13 (blue) at the antigen–antibody interface within a distance of 3.5 Å.

The cryo-EM reconstruction of CODV_Ig_:IL13:RefAb_Fab_ (Fig. 2a) evinces electron density for CODV_Fab_, IL13 and RefAb_Fab_. With an overall resolution of 4.2 Å (Fig. 2b), the CODV_Fab_:IL13 and IL13:RefAb_Fab_ epitope-paratope regions exhibit the highest resolution while the IL4 binding subdomains of CODV (VH2 and VL2) and both C1 subdomains of CODV and RefAb, located away from the IL13 binding regions, are at more modest resolutions, in the 6–7 Å range (Figs. 2c and S7). Density for the L3 linker region, connecting VH2 to VH1 and for L4 connecting VH1 to CH1, have better defined densities compared to those observed for the L1 and L2 linkers which connect VL1 to VL2 and VL2 to CL, respectively.

**Figure 3:**
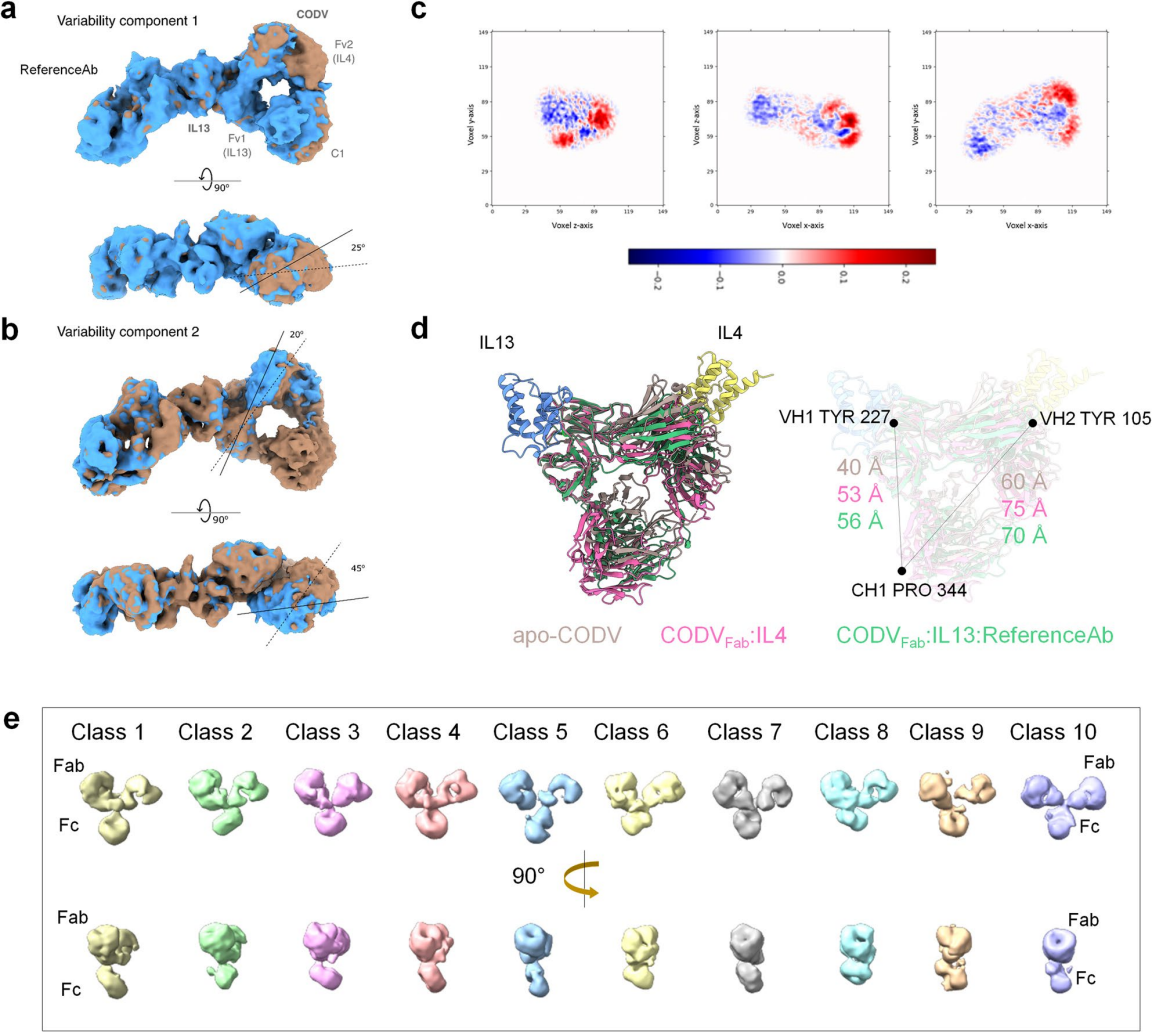
Flexibility analyses of Fab and Fc domains of CODV_Ig_. (**a**) and (**b**), The two components resulting from the 3D variability analysis (3DVA) of the TC map indicating the high flexibility present in IL4-binding and constant domains. Clockwise rotation for anti-IL4 Fv2 is observed in the variability component 1 whereas, as shown in (**b**), variability component 2 exhibits a counter-clockwise movement of the anti-IL4 Fv2. The first frame of the 3DVA volume series is in blue, while the last frame is in orange. These results are also shown as a “supplementary video". (**c**) Slices along planes of the three orthogonal axes are shown for the variability component 2 shown in b, with blue and red colors represent density to be added and subtracted from the mean density indicating the movement in either direction along the axes. (**d**) Superposition of the crystal structures of *apo*-CODV_Fab_, CODV_Fab_:IL4 and the cryo-EM structure of TC (this work) showing the movement of the constant domain in the antigen bound structures away from the antigen sites. This antigen induced conformational change is proposed to be transferred to the Fc region via the Fab-Fc hinge inducing additional flexibility to Fc. (**e**) Different classes from the 3D classification of CODV:IL4 indicating different structural dispositions of Fc relative to the Fabs.

Although the estimated local resolution of the cryo-EM map at the CODV_Fab_:IL13 binding interface is better than that of the rest of the molecule (Fig. 2c) it is, nevertheless, still moderate. PDBePISA^16^ was thus used to predict atom–atom interactions, the amino acid residues involved in this interface and putative CODV:IL13 H-bonds. These were then confirmed in the cryo-EM density. The reconstruction obtained shows that all 3 CDR loops of CODV VH1 and CDR1 and CDR3 of VL1 are in close proximity to IL13 with higher buried surface area (BSA) compared to rest of the molecule (Fig. 2d and Table S3). Interestingly, CDR2 of VL1 (Leu-Ala-Ser), exhibits the least buried surface area (BSA) and is not involved in any H-bond interactions with IL13. The heavy chain VH1 shares twice the interface area (564 Å^2^) with IL13 compared to VL1. Within VH1, CDR3 participates strongly with a maximum BSA of 312Å^2^ while CDR1 interacts the least (BSA of 92 Å^2^). Hydrogen bond interactions follow this trend (Table 1 and Fig. 2e). Here, Asp155_CDR1_ and Ser156_CDR1_ interact with Ser73_IL13_, Arg180_CDR2_ forms hydrogen bonds with Ser76_IL13_ and Tyr227_CDR3_ hydrogen bonds with Val72_IL13_, Gly74_IL13_ and Gln82_IL13_. VL1 makes only three potential hydrogen bonds with IL13, namely Gln34_CDR1_-Arg83_IL13_, Gln34_CDR1_-Ser86_IL13_ and Asn95_CDR3_-Arg83_IL13_. All main chain atoms of these interface residues have well-defined EM densities as do all side chains except Gln34_CDR1_, Asn95_CDR3_ and Asp155 _CDR1_ (Fig. 2e). For these latter residues, the side chains have been oriented according to the model refinement criteria. Notably, there are no potential salt-bridges between IL13 and CODV_Fab_, contrary to what has been observed for IL4 binding to CODV_Fab_ in the crystal structure of CODV_Fab_:IL4^11^. This observation of a lower number of CODV_Fab_:IL13 interactions compared to CODV_Fab_:IL4 is commensurate with the lower affinity of CODV_Ig_ for IL13. Importantly though, our reconstruction shows that the CODV_Ig_ IL13 binding site (Fv1) is oriented ~ 90° degrees to that of IL4 (Fv2), with the relevant antigen binding sites more than 50 Å apart (Fig. 3d). This suggests the binding of either of the antigen to CODV_Fab_ is not restricted by the presence or absence of the other antigen.

**Table 1:** Putative hydrogen bond interactions between CODV_Fab_ and IL13 computed by PDBePISA server.

Heavy chain (VH1)	IL13	Distance in Å
ASP 155[O]	SER 73[OG]	2.4
SER 156[OG]	SER 73[OG]	3.8
ARG 180[NH1]	SER 76[OG]	3.7
TYR 227[OH]	GLY 74[O]	2.8
TYR 227[OH]	VAL 72[O]	3.0
TYR 227[OH]	GLN 82[NE2]	3.8

Light chain (VH1)	IL13	Distance in Å
ASN 95[O]	ARG 83[NH2]	3.2
GLN 34[NE2]	ARG 83[O]	3.7
GLN 34[NE2]	SER 86[OG]	2.5

In addition to providing previously unknown details of CODV_Ig_:IL13 interactions, our reconstruction confirms details of IL13:RefAb_Fab_ paratope-epitope interactions previously observed in the crystal structure of IL13:RefAb_Fab_^15^. The side chains of most of the residues involved at this interface are in the same orientation as in the crystal structure and fit well in the EM density (Fig. S7). The presence of CODV_Ig_ has thus not perturbed the interaction of IL13 with RefAb_Fab_.

### IL13 binding induces flexibility in the Fab and constant domains of CODV_Ig_

The relative orientation of variable and constant domains in the Fab allosterically dictates the structural disposition of the Fc domain via the Fab-Fc hinge region. It is thus of critical importance to know the effect of antigen binding on the orientation of these domains as Fc controls the antibody's effector response^9,11^. We therefore investigated if any conformational changes are induced upon RefAb_Fab_:IL13 binding to CODV_Fab_, particularly in comparison to the reported crystal structures of *apo-*CODV_Fab_^11^ and CODV_Fab_:IL4^11^.

In the cryo-EM reconstruction of CODV_Fab_:IL13:RefAb, the lower resolutions observed away from the CODV_Fab_:IL13 and IL13:RefAb epitope-paratope interfaces are clearly related to the high inter-domain flexibility allowed by the linker regions of the CODV format (Fig. 2c). Principal component analysis^17^ suggests rotations and translations of the IL4 binding subdomains of CODV and of the C1 subdomains of CODV and RefAb, while the CODV_Fab_:IL13 interface, flanked by the two anti-IL13 Fv domains, remains relatively rigid (Fig. 3a–c and Supplementary Video 1). In the movements analysed, the IL4-binding fragment, Fv2 presents a 25° clockwise rotation on the Y axis (Fig. 3a—variability component 1), while the linker, L2, connecting the light chains of C1 and IL4-binding Fv2, seems to allow a small 8 Å outwards translation of C1 resulting in Fv2 moving away from Fv1. In the other major movement observed (Fig. 3b—variability component 2), Fv2 shows an approximately 20° clockwise rotation on the Z axis relative to the IL-13 bound Fv1 and a 45° counter-clockwise rotation around Y axis, further extending the distance and space between the two antigen-binding domains and their binding sites. The observed widening of the IL4 and IL13 paratopes and the intrinsic flexibility and interconnectivity provided by the linkers may aid CODV in rearranging its Fvs, thus facilitating binding of larger ligands, such as HER2 and HER3 located in the cellular membrane, as hypothesised in previous CODV-related work^11^.

In line with the above observation concerning the mobility of CODV_Ig_ C1, a superposition of the cryo-EM reconstruction of CODV_Fab_:IL13:RefAb_Fab_ with the crystal structures of *apo-*CODV_Fab_ and CODV_Fab_:IL4 on Fv1 domain indicates that, upon the binding of IL13:RefAb_Fab_, the constant domain moves away from both antigen binding sites (Fig. 3d). Upon binding, each antigen Fab seems to push C1 away from the respective variable domains. This movement could be transferred to the Fc domain via the hinge that connects the C1 to the Fc^18,19^. In support of this, in our low resolution cryo-EM reconstructions of the full CODV_Ig_:IL4, the electron density for Fc is positioned at different orientations relative to the Fab regions (Fig. 3e).

## Discussion

**Figure 4:**
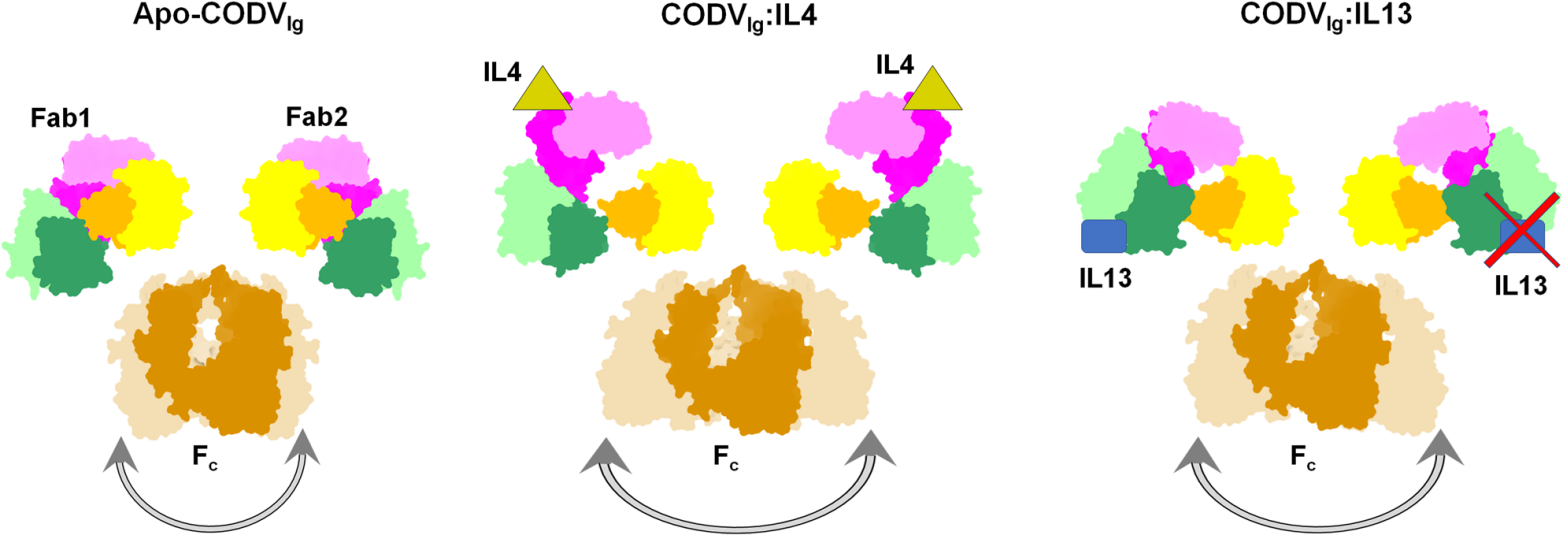
Model explaining the observed 1:1 stoichiometry for IL13: antigen CODV_Ig_: The structural flexibility of Fc domain evident from our cryo-EM studies of full antibody CODV_Ig_ results in an inclination of the Fc domain relative to the Fab domains. This would occlude, depending on Fc orientation, one of the CODV_Ig_ binding sites. However, it cannot be discounted that structural rearrangements in the Fab domains, induced due to antigen binding (IL4 or IL13), can be translated to Fc domain via the hinge region to induce additional flexibility to Fc, which, for reasons yet unknown, hinders the binding of IL13 to both Fabs simultaneously.

Atomic resolution details, including the relative positions of different domains, in particular the variable domains, and their conformations, of the structure of full antibody CODV_Ig_ in complex with IL4 and IL13 will be of valuable information in understanding the CODV_Ig_ framework’s capability to simultaneously bind to these two antigens. Existing structural studies include the crystal structures of both *apo-*CODV_Fab_ and CODV_Fab_:IL4^11^. However, attempts to crystallize CODV_Fab_:IL13 or CODV_Fab_:IL4:IL13 have not yet been successful. We therefore pursued a cryo-EM approach, investigating the structure of CODV_Ig_:IL13, in a full antibody context, as a precursor to the potential study of CODV_Ig_:IL4:IL13. The high level of flexibility of the CODV Fc domain means we have only been partially successful, resorting to a combination of labelling and a ‘Fab-focused’ approaches to produce a cryo-EM reconstruction of a CODV_Fab_:IL13:RefAb_Fab_. The results of our structural analysis are intriguing: they clearly suggest that the disposition of the IL4 and IL13 Fab domains should allow the binding of both antigens at the same time to the same Fab. Moreover, our results did not provide a direct suggestion why CODV_Ig_ binds IL13 primarily with a 1:1 stoichiometry rather than the 1:2 ratio observed for CODV_Ig_:IL4. However, a possible explanation may be found both in our low resolution cryo-EM analysis of CODV_Ig_:IL4 and CODV_Ig_:IL4:IL13 complexes (Fig. S2) and in the crystal structures of anti-canine lymphoma Mab231, an IG2a antibody (PDB 1IGT^18^) and of anti-pheno-barbital (anti-epileptic drug) Mab 61.1.3, an IG1k antibody (PDB 1IGY^19^). Both in the crystal structures reported and in some of the low resolution classes we observe for CODV_Ig_:IL4 and CODV_Ig_:IL4:IL13 complexes (Figs. 3e and S2), the Fc domain is observed in an “inclined” disposition leading to a non-symmetrical interaction of Fc with respect to the Fab fragments. We propose that this propensity of Fc to assume an oblique disposition to one of the Fabs may lead to occlusion of the IL13 binding site in that Fab (Fig. 4).

Additionally, the crystal structures of full Igs have been proposed to be one of the several structural states that the antibody can assume due to the prevailing internal mobility. As also observed in the structure of Mab231 and elsewhere^20,21^, the Fc domain of CODV_Ig_ also exhibits a substantial degree of internal mobility represented by various classes in 3D classification (Figs. S2 and S6) and due to this fact it was mostly ghosted out in 2D classification. It is not still clear whether this fluctuation in the structural disposition of Fc could be translated to Fab fragments or vice versa with or without antigen binding. Nevertheless, when we compare the domain movements within Fab induced by antigen binding (IL4 or IL13), we observe significant movement of C1 domain relative to the Fv1 and Fv2 domains (Fig. 3d). Whether these antigen-induced movements in the C1 domains of each Fab have a structural influence or place constraints on the disposition of Fc (Fig. 4) requires further investigation. This would require the full antibody structure, perhaps with Fc bound to a third protein and/or with deletion mutants of hinge region. Also, further investigation is necessary to ascertain whether the structural flexibility of Fc and/or the motion observed in the C1 domain can have an effect on the existence of fewer interactions at the epitope-paratope region of CODVFab:IL13 compared to CODVFab:IL4, as fewer protein–protein interactions and subsequently lower affinity could lead to a lower stoichiometry.

In the cryo-EM structure of CODV_Fab_:IL13:RefAb_Fab_ and in all the cryo-EM reconstructions shown here, the segmental flexibility of Fc in relation to Fab fragments has been routinely observed. It is worth mentioning that the only attempt thus far to obtain structural information using cryo-EM on an intact full bispecific antibody is that for the DVD-Ig_IL12XIL18_. Here also, the Fc exhibited huge flexibility such that its density was not observed in many class averages^12^. Although crystal structures exist for intact IgG1 and IgG2 antibodies, they represent a single snapshot from the several structural states the antibodies could adopt^18,19^. Furthermore, several other crystal structures of intact IgG molecules show disordered Fc domains^22^. The only way to restrain this flexibility is to delete the hinge region^23^. Indeed, the length of hinge regions is associated strongly with the available segmental flexibility in an antibody^24–26^. However, alterations in hinge length have notable effects on the important effector functions of Fc such as receptor binding, complement activation, etc^23,26–30^.

Owing to the high affinity of IL4 and IL13 for CODV_Ig_, we believe that it is unlikely that the ligands separate from the antibody in our experimental conditions and attribute the difficulty in their visualisation by cryo-EM to the small size of the ligand and intrinsic flexibility of the antibody. Attaching RefAb to IL13 seems to limit this flexibility, as evidenced by our 3D classification results. Nevertheless, more experiments, particularly medium- to high-resolution structural studies of the CODV_Ig_:IL13:IL4 complex, are required to confirm the stoichiometry of IL13 binding to CODV_Ig_. Despite obtaining stable CODV_Ig_:IL13:IL4:RefAb by gel filtration, structural studies by EM are currently unsuccessful and the identification of a suitable antibody label for IL4 may be needed.

In conclusion, the work presented here has shown that it is indeed possible to use cryo-EM as a tool for the medium resolution visualisation multi-specific antibody-antigen interfaces within the full-antibody context. However, owing to the intrinsic flexibility of the Fab and Fab-Fc regions, the binding of the small antigens IL4 and IL13 could not be directly imaged either in nsEM or in cryo-EM reconstructions. Indeed, such imaging requires antigen labelling with a molecule of which the binding does not interfere with antibody-antigen binding sites. Here we have used secondary antibody labelling in the cryo-EM study of a multi-specific antibody-antigen complex and this may prove a general approach. Indeed, a similar method has been recently exploited in cryo-EM studies of membrane proteins. Here a synthetic Fab (NabFab) has been used as a label for the small 15 kDa nanobody bound to a membrane protein, thus enhancing the molecular weight to facilitate the cryo-EM analysis^31^. In summary, our cryo-EM reconstruction of CODV_Ig_:IL13:RefAb_Fab_ ternary complex in the full antibody context confirms that CODV_Ig_ is structurally competent for the simultaneous binding of the interleukins IL4 and IL13 and therefore remains a very promising platform for cancer immunotherapy. The reconstruction appears to confirm a preference, observed in solution, for a 1:1 CODV_Ig_:IL13 stoichiometry. The study also provides the first visualisation of CODV_Ig_:IL13 paratope-epitope interactions, providing a structural platform to enhance the therapeutic potential of CODV_Ig_. Finally, the use of labelling with a secondary antibody to reduce the flexibility of the CODV_Fab_:IL13 region of the ternary complex and to facilitate FAB-focused particle alignment in cryo-EM analysis suggests this could be used as a general approach for the cryo-EM analysis of full-antibody msAbs. Such an approach has been an important tool for the success of cryo-EM analyses of inherently flexible molecules of high therapeutic interest.

## Materials and methods

### Preparation and purification of antibodies and antibody-antigen complexes

Cloning procedures for all constructs are explained elsewhere^11^. CODVIg_IL4 x IL1 3_ (CODV_Ig_) was from Human Embrionic Kidney (HEK 293) cells (Invitrogen) and was purified at Sanofi-Aventis Deutschland Gmbh (Frankfurt-am-Main, Germany), as described in^11^. His-tagged IL4 and IL13 proteins were also expressed in HEK 293 cells (Life technologies) and purified at Evotec (France) SAS, by two-step procedure using His-Trap and SEC. Preparations of *apo-*CODV_Ig_ were stored in *His buffer* (Histidine 10 mM, NaCl 150 mM, pH 6.0), IL4 and IL13 were on *DPBS* (5 mM Phosphate, 155 mM NaCl, pH 7.4) and RefAb_Fab_ samples were in *TBS* (20 mM Tris, 50 mM NaCl, pH 8.0).

For complexes containing only IL4 and/or IL13, these were formed by adding fourfold molar excess of ligand(s) to 1 mg/ml of CODV_Ig_. CODV_Ig_:IL4, CODV_Ig_:IL13 and CODV_Ig_:IL4:IL13 complexes were purified via size exclusion chromatography (SEC) using a Superdex S200 3.2/300 Increase column equilibrated with *SB buffer* (50 mM Tris, 95 mM NaCl, 3 mM KCl, pH 8.0) on an ÄKTA Pure (GE Healthcare) at 4 °C. Fractions were collected with an ÄKTA Micro module (GE Healthcare) and those containing CODV_Ig_:IL4, CODV_Ig_:IL13 and CODV_Ig_:IL4:IL13 did not require concentration steps, as they eluted at concentrations in the range of 0.25 to 0.30 mg/ml, suitable for direct grid preparation for cryo-EM. The purified complexes were stored at 4 °C.

For the purification of the CODV_Ig_:IL13:RefAb_Fab_ ternary complex (TC), IL13 was first added in fourfold molar excess to CODV_Ig_ at 1 mg/ml and incubated for 15 min. RefAb_Fab_ was then added, also in fourfold molar excess. For purification, a single high-resolution SEC step was performed as outlined above for CODVIg and its complexes with IL14 and/or IL13. As in all the other complexes, only the fractions corresponding to the centre of the peak were collected for cryo-EM grid preparation. The purified ternary complex was stored at 4 °C.

Throughout all purification procedures, eluted fractions were analysed using denaturing 15% sodium dodecyl sulphate polyacrylamide gel electrophoresis (SDS-PAGE). Protein concentration was checked using a Nanodrop ND-1000 spectrophotometer (Thermo Fisher Scientific) in combination with the theoretical absorption coefficients calculated using the PROTPARAM online tool provided by ExPASy^32^.

### Characterization of antigen–antibody complexes by mass photometry

All samples were subjected to analysis by mass photometry^33^. Here, 100 nM of CODV_Ig_, CODV_Ig_:IL4, CODV_Ig_:IL13, CODV_Ig_:IL4:IL13 and CODV_Ig_:IL13:RefAb_Fab_ were analysed using a OneMP mass photometer (Refeyn Ltd, Oxford, UK), previously calibrated with *SB buffer* to find the focal point and to estimate background noise. Subsequent to the measurements, image processing was performed as described in Sonn-Segev et al.^33^ using the DiscoverMP (Refeyn Ltd) software package and graphs of intensity vs molecular weight plotted.

### Characterization of antigen–antibody complexes by SPR

Antigen binding and affinity parameters for CODV_Ig_:IL4 and CODV_Ig_:IL13 were measured by Surface Plasmon Resonance (SPR) in a Biacore 8 K SPR system (GE Healthcare). A dextran CM5 sensor chip was used for the protocol, which immobilized anti-human IgG-Fc overnight via amine-coupling. Flow-cell 1 (FC1) was used as a reference while FC2 was utilized for CODV_Ig_ and the ligands. The buffer used for the runs was *STBS* (50 mM Tris, 50 mM NaCl, pH 8) with the addition of Tween 20 at 0.01% to lower the surface tension and reduce non-specific binding.

For the evaluation of the dissociation constants (KD) of IL4 and IL13 binding to CODV, several runs were performed with serial dilutions of the antigens. Following the determination of the binding parameters, final SPR runs were carried out with concentrations of 1.5 µg/ml (7.6 nM) CODV_Ig_, 3 nM IL4 and 25 nM IL13.

### Structural assessment by small angle X-ray scattering

Small Angle X-Ray Scattering (SAXS) measurements of gel-filtration purified CODV_Ig_:IL13:RefAb_Fab_ were obtained using X-rays of 12.5 keV energy (λ = 0.99 Å) and a Pilatus 1 M pixel array detector (Dectris) at the ESRF BioSAXS beamline, BM29^34^. 3600 individual frames with a collection time 1 s per frame were collected. Of these, 183 frames were selected, chosen using the similarity plot in ScatterIV^35^. These were merged and subtracted from the buffer. Primary data analysis was carried out using ScatterIV and low-resolution envelopes of the solution structures obtained using DAMMIF from ATSAS^36^ online tool from EMBL Hamburg. 100 µl of freshly purified TC was used at a concentration of 1 mg/ml in the SEC-SAXS variant of the technique^37^. The column and flow rate used were identical to those used in the purification of TC (Superose 6 Increase 3.2/300 at 0.04 ml/min), while the chromatography system was a Nexera X2 HPLC (Shimadzu) and the buffer used was *SB* with the addition of 1 mM tris(2-carboxyethyl)phosphine (TCEP) to protect the sample from radiation damage^38^. Other parameters are listed in Table S4.

### nsEM Grid preparation

Of the various stains tried, 2% SST was found to be the most suitable for CODV_Ig_ and the complexes studied here. For sample preparation, 3 µl of a solution containing antibody or antibody-antigen complexes was adsorbed to the interface between carbon and mica which, after few seconds of incubation, was slowly inserted into a well that contains the stain. A clean 400-mesh copper grid was placed on the carbon and the whole carbon-grid assembly air-dried. For good particle distribution, optimal sample concentrations were observed to be around 10 µg/ml for CODV_Ig_-antigen complexes and 15 µg/ml for *apo-*CODV_Ig_ alone.

### nsEM data collection and image processing

nsEM grids were screened at the Grenoble Institut de Biologie structurale (IBS) Electron Microscope facility using a Tecnai T-12 120 keV transmission electron microscope (Thermo Fisher Scientific) fitted with a Gatan Orius SC 600 camera (AMETEK). Data collection for image analysis was performed using a Tecnai F-20 200 keV microscope equipped with a 4 K x 4 K Ceta CMOS camera (Thermo Fisher Scientific). Micrographs were recorded using the TEM Imaging & Analysis (TIA, Thermofisher Scientific) software under low-dose mode.

Estimation of CTF parameters was carried out using CTFFIND4^39^. Particles were picked fully automatically using the Laplacian or Gaussian (LoG) blob detection algorithm implemented in RELION 3.0^40^, and further 2D and 3D classifications were also performed using RELION 3.0.

### Cryo-EM sample preparation and data collection

3 µl of the samples at a 300 μg/ml concentration supplemented with 0.03 mM DDM were applied to an Au-CFlat 1.2/1.3 300 mesh grid and vitrified using a Vitrobot (ThermoFisher Scientific) using a blot time of 7 s and a blot force of 2.

Grid optimisation screening was performed at the IBS Electron Microscope facility using a 200 keV Glacios microscope equipped with both Falcon II detector (Thermo Fisher Scientific) and a Gatan K2 detector (AMETEK). All high-resolution data collections were performed on the ESRF CM01 beamline^41^ using a 300 keV Titan Krios Microscope (Thermo Fisher Scientific) equipped with a 4 k x 4 k Gatan K2 Summit direct electron detector and a Gatan Bioquantum LS/967 energy filter (AMETEK). Either EPU (Thermo Fisher Scientific) or SerialEM was used for automatic image acquisition.

The CODV_Ig_:IL13:RefAb_Fab_ untilted dataset (14,010 movies) was collected at a nominal magnification of 165000X corresponding to a pixel size of 0.827 Å and a total dose of 46 e^-^/Å^2^. The defocus values range between −1.2 and −3.0 μm. The CODV_Ig_:IL13:RefAb_Fab_ tilted dataset (1188 movies), was performed exploiting a 40° stage tilt and a total dose of 52 e^−^/Å^2^.

The CODV_Ig_:IL4 dataset comprised 7044 movies collected over a defocus range of -1.5 to -3.5 μm and a total dose of ~ 60 e^−^/Å^2^ and a pixel size of 0.827 Å. The CODV_Ig_:IL4:IL13 dataset (2919 movies) was collected using a similar defocus ranges and total dose, with a pixel size 1.053 Å.

### Cryo-EM image processing and 3D reconstruction

The datasets for CODV_Ig_:IL13:RefAb_Fab_, CODV_Ig_:IL4 and CODV_Ig_:IL4:IL13 were processed until the 2D classification step within the cryoSPARC2^42^ environment. Motion correction and dose weighting of movies was carried out using MotionCor2^43^ while CTF estimation was carried out with the Patch CTF functionality of cryoSPARC2.

For all three data sets, initial manual particle picking was followed by an automatic protocol using the class averages from the manual step as templates (cryoSPARC2). Suitable particles identified from 2D classification were then imported into RELION 3.0 classification. For particles used in the Fab-focused refinement, 2D class averages from the full-antibody processing pipeline were first centered on the Fab density using a 110 Å mask and a high center-of-mass fraction value for recentering. The centered Fabs were then used as templates for automatic repicking of Fab densities. 2D classification in cryoSPARC2 was used to separate high-resolution classes and corresponding particles were imported further into RELION 3 for the 3D processing. In RELION 3.0, *ab-initio* model generation and 3D classification rounds were performed with the aim of isolating single conformations and classes that could contain densities corresponding to antigens. Finally, 3D multivariance analysis of TC was performed using cryoSPARC3^17^.

Bayesian polishing was performed within RELION 3.1. The global resolution of the final map of all the reported structures was estimated according to the gold standard Fourier shell correlation (GS-FSC) with a cutoff of 0.143^44^. Local resolution variability was estimated using ResMap^45^, with half-maps as input volumes.

EM density maps obtained were visualized with UCSF Chimera^46^ and published structures of apo-CODV_Fab_ (PDB code: 5HCG^11^) and the knob in hole IgG-Fc (PDB code: 4NQS^47^) were used for docking.

### Model building and refinement

Models for fitting and refinement were obtained by homology modelling using SWISS-MODEL^48^, based on NCBI BlastP^49^ alignment between the sequences of the proteins used for this study and the already published individual PDB structures of IL13:RefAb_Fab_ (PDB code: 5L6Y^15^) and apo-CODV_Fab_. Rigid-body fitting of the model to map was performed using Chimera and further refined by positional refinement using Real Space Refine within Phenix^50^. Map sharpening was performed either using LocScale^51^ from the CCPEM^52^ package or Autosharpen within Phenix^50^. Manual model building and validation were performed in Coot^53^ and Molprobity^54^ respectively. EM data collection parameters and refinement statistics for the TC are presented in Table S2.

## Supplementary Information

Supplementary Information.

Supplementary Information.

## Data Availability

The cryo-EM reconstruction and the 3D model of CODV_Fab_:IL13:RefAb have been deposited in the EM Data Bank (https://www.ebi.ac.uk/emdb/) and PDB data bank (https://www.rcsb.org) with the accession codes EMD-16113 and 8BLQ, respectively. All data needed to evaluate the conclusions in the paper are present in the paper or the Supplementary Materials. Request for materials will be subject to a standard material transfer agreement with Sanofi.

## References

[1] Brinkmann U, Kontermann RE (2017). The making of bispecific antibodies. mAbs.

[2] Labrijn AF, Janmaat ML, Reichert JM, Parren PWHI (2019). Bispecific antibodies: A mechanistic review of the pipeline. Nat. Rev. Drug Discov..

[3] Husain B, Ellerman D (2018). Expanding the boundaries of biotherapeutics with bispecific antibodies. BioDrugs.

[4] Panke C (2015). Bi- and Multi-specific Antibodies.

[5] Brinkmann U, Kontermann RE (2021). Bispecific antibodies. Science.

[6] Weidanz J (2021). Targeting cancer with bispecific antibodies. Science.

[7] Godar M, de Haard H, Blanchetot C, Rasser J (2018). Therapeutic bispecific antibody formats: A patent applications review (1994–2017). Expert Opin. Ther. Pat..

[8] Kreudenstein TSV (2013). Improving biophysical properties of a bispecific antibody scaffold to aid developability: Quality by molecular design. mAbs.

[9] Lee CH (2017). IgG Fc domains that bind C1q but not effector Fc3 receptors delineate the importance of complement-mediated effector functions. Nat. Immunol..

[10] Li T (2017). Modulating IgG effector function by Fc glycan engineering. Proc. Natl. Acad. Sci. U.S.A..

[11] Steinmetz A (2016). CODV-Ig, a universal bispecific tetravalent and multifunctional immunoglobulin format for medical applications. mAbs.

[12] Jakob CG (2013). Structure reveals function of the dual variable domain immunoglobulin (DVD-Ig^TM^) molecule. mAbs.

[13] Viganò E (2018). Somatic IL4R mutations in primary mediastinal large B-cell lymphoma lead to constitutive JAK-STAT signaling activation. Blood.

[14] Rawlings JS, Rosler KM, Harrison DA (2004). The JAK/STAT signaling pathway. J. Cell Sci..

[15] Popovic B (2017). Structural characterisation reveals mechanism of IL-13-neutralising monoclonal antibody tralokinumab as inhibition of binding to IL-13Rα1 and IL-13Rα2. J. Mol. Biol..

[16] Krissinel E, Henrick K (2007). Inference of macromolecular assemblies from crystalline state. J. Mol. Biol..

[17] Punjani A, Fleet DJ (2021). 3D variability analysis: Resolving continuous flexibility and discrete heterogeneity from single particle cryo-EM. J. Struct. Biol..

[18] Harris LJ, Larson SB, Hasel KW, McPherson A (1997). Refined structure of an intact IgG2a monoclonal antibody. Biochemistry.

[19] Harris LJ, Skaletsky E, McPherson A (1998). Crystallographic structure of an intact IgG1 monoclonal antibody. J. Mol. Biol..

[20] Colman PM, Deisenhofer J, Huber R, Palm W (1976). Structure of the human antibody molecule kol (immunoglobulin G1): An electron density map at 5 Å resolution. J. Mol. Biol..

[21] Ely KR (1978). Mobile Fc region in the Zie IgG2 cryoglobulin: Comparison of crystals of the F(ab’)2 fragment and the intact immunoglobulin. Biochemistry.

[22] Marquart M, Deisenhofer J, Huber R, Palm W (1980). Crystallographic refinement and atomic models of the intact immunoglobulin molecule Kol and its antigen-binding fragment at 3.0 Å and 1.9 Å resolution. J. Mol. Biol..

[23] Guddat LW, Herron JN, Edmundson AB (1993). Three-dimensional structure of a human immunoglobulin with a hinge deletion. Proc. Natl. Acad. Sci..

[24] Yguerabide J, Epstein HF, Stryer L (1970). Segmental flexibility in an antibody molecule. J. Mol. Biol..

[25] Klein M (1981). Expression of biological effector functions by immunoglobulin G molecules lacking the hinge region. Proc. Natl. Acad. Sci. U.S.A..

[26] Oi VT (1984). Correlation between segmental flexibility and effector function of antibodies. Nature.

[27] Deutsch HF, Suzuki T (1971). A crystalline γg1 human monoclonal protein with an excessive h chain deletion. Ann. N. Y. Acad. Sci..

[28] Fett JW, Deutsch HF, Smithies O (1973). Hinge-regIon deletion localized in the IgG1-globulin Mcg. Immunochemistry.

[29] Burton DR (1985). Immunoglobulin G: Functional sites. Mol. Immunol..

[30] Isenman DE, Dorrington KJ, Painter RH (1975). The structure and function of immunoglobulin domains. II. The importance of interchain disulfide bonds and the possible role of molecular flexibility in the interaction between immunoglobulin G and complement. J. Immunol..

[31] Bloch JS (2021). Development of a universal nanobody-binding Fab module for fiducial-assisted cryo-EM studies of membrane proteins. Proc. Natl. Acad. Sci..

[32] Gasteiger E (2005). Protein identification and analysis tools on the ExPASy server. The Proteomics Protocols Handbook.

[33] Sonn-Segev A (2020). Quantifying the heterogeneity of macromolecular machines by mass photometry. Nat. Commun..

[34] Pernot P (2013). Upgraded ESRF BM29 beamline for SAXS on macromolecules in solution. J. Synchrotron. Rad..

[35] Tully MD, Tarbouriech N, Rambo RP, Hutin S (2021). Analysis of SEC-SAXS data via EFA deconvolution and Scatter. JoVE.

[36] Franke D (2017). ATSAS 2.8: A comprehensive data analysis suite for small-angle scattering from macromolecular solutions. J. Appl. Crystallogr..

[37] Watanabe Y, Inoko Y (2009). Size-exclusion chromatography combined with small-angle X-ray scattering optics. J. Chromatogr. A.

[38] Jeffries CM, Graewert MA, Svergun DI, Blanchet CE (2015). Limiting radiation damage for high-brilliance biological solution scattering: Practical experience at the EMBL P12 beamline PETRAIII. J. Synchrotron Radiat..

[39] Rohou A, Grigorieff N (2015). CTFFIND4: Fast and accurate defocus estimation from electron micrographs. J. Struct. Biol..

[40] Zivanov J (2018). RELION-3: New tools for automated high-resolution cryo-EM structure determination. bioRxiv.

[41] Kandiah E (2019). CM01: A facility for cryo-electron microscopy at the European synchrotron. Acta Crystallogr. Sect. D: Struct. Biol..

[42] Punjani A, Rubinstein JL, Fleet DJ, Brubaker MA (2017). cryoSPARC: Algorithms for rapid unsupervised cryo-EM structure determination. Nat. Methods.

[43] Zheng SQ (2017). MotionCor2: Anisotropic correction of beam-induced motion for improved cryo-electron microscopy. Nat. Methods.

[44] Rosenthal PB, Henderson R (2003). Optimal determination of particle orientation, absolute hand, and contrast loss in single-particle electron cryomicroscopy. J. Mol. Biol..

[45] Kucukelbir A, Sigworth FJ, Tagare HD (2014). Quantifying the local resolution of cryo-EM density maps. Nat. Methods.

[46] Pettersen EF (2004). UCSF Chimera—A visualization system for exploratory research and analysis. J. Comput. Chem..

[47] Elliott JM (2014). Antiparallel conformation of knob and hole aglycosylated half-antibody homodimers is mediated by a CH2–CH3 hydrophobic interaction. J. Mol. Biol..

[48] Waterhouse A (2018). SWISS-MODEL: Homology modelling of protein structures and complexes. Nucleic Acids Res..

[49] Johnson M (2008). NCBI BLAST: a better web interface. Nucleic Acids Res..

[50] Afonine PV (2018). Real-space refinement in PHENIX for cryo-EM and crystallography. Acta Crystallogr. Sect. D: Struct. Biol..

[51] Jakobi AJ, Wilmanns M, Sachse C (2017). Model-based local density sharpening of cryo-EM maps. eLife.

[52] Wood C (2015). Collaborative computational project for electron cryo-microscopy. Acta Crystallogr. D Biol. Crystallogr..

[53] Emsley P, Cowtan K (2004). Coot: Model-building tools for molecular graphics. Acta Crystallogr. D Biol. Crystallogr..

[54] Williams CJ (2018). MolProbity: More and better reference data for improved all-atom structure validation. Protein Sci..

