# Supplementary material for: Structural insights into the bi-specific cross-over dual variable antibody architecture by cryo-EM
Source: Sci Rep. 2023 May 29;13:8694. doi: 10.1038/s41598-023-35678-4 (PMC10227088; doi:10.1038/s41598-023-35678-4)
Supplement: Supplementary file 1 — Supplementary Information. [file 41598_2023_35678_MOESM1_ESM.docx]

Title: Structural insights into the bi-specific cross-over dual variable antibody architecture by cryo-EM

**Authors:** David Fernandez-Martinez^1,2,3^, Mark D Tully^1^, Gordon Leonard^1^, Magali Mathieu^2*^**,** Eaazhisai Kandiah^1*^

**Affiliations:**

^1^ European Synchrotron Radiation Facility; 71 Avenue des Martyrs, 38042 Grenoble, France

^2^ Sanofi R&D, Bio Structure and Biophysics, Centre de Recherche Vitry-sur-Seine ; 94403 Vitry-sur-Seine Cedex, France

^3^ Current address: Pathogenesis of Vascular Infections, Department of Cell Biology and Infection, Institut Pasteur, INSERM, 75015 Paris, France.

*Corresponding authors. Email: [Magali.Mathieu@sanofi.com](mailto:Magali.Mathieu@sanofi.com); [eaazhisai.kandiah@esrf.fr](mailto:eaazhisai.kandiah@esrf.fr)

**SUPPLEMENTARY FIGURES**


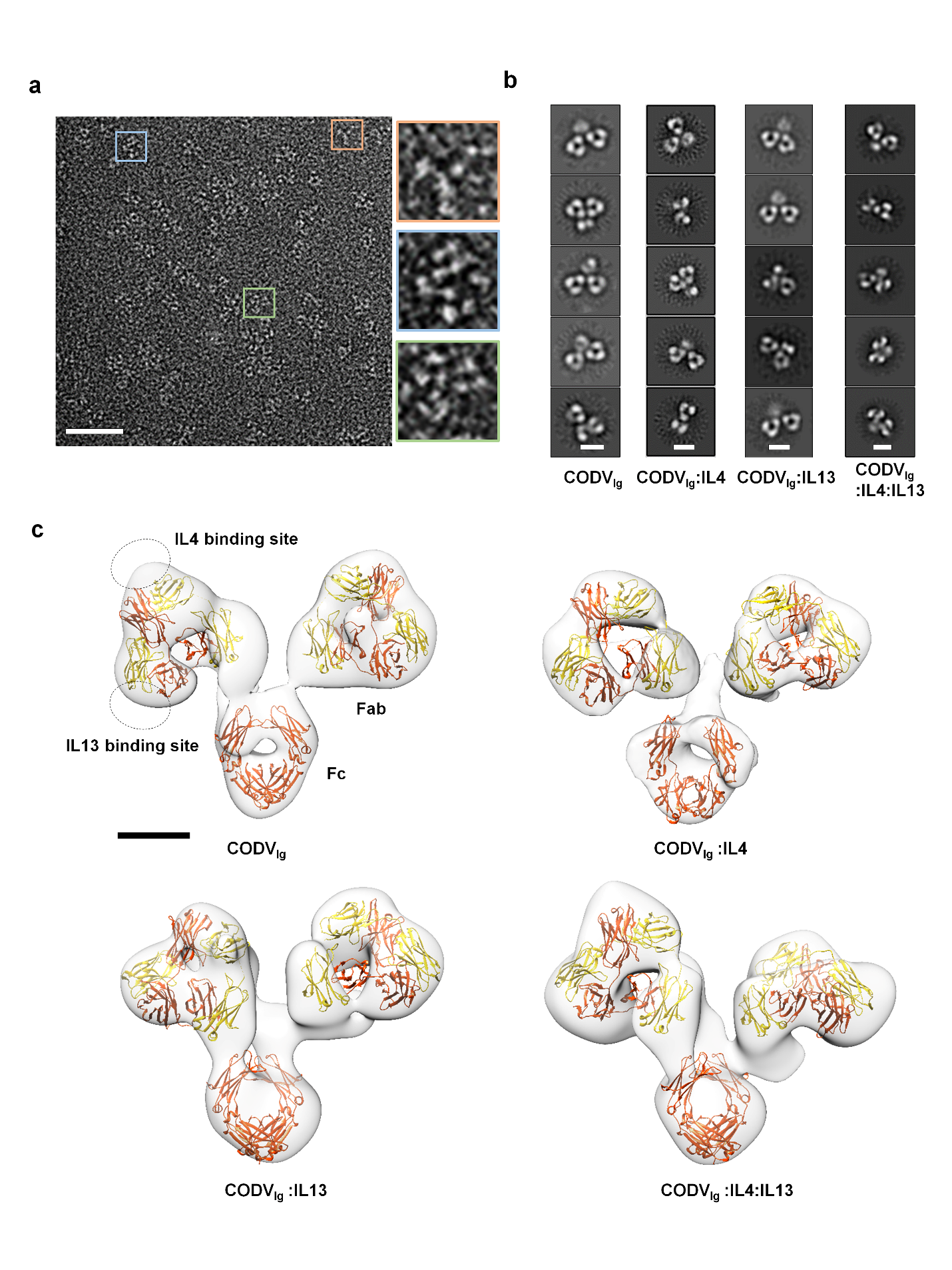


**Fig. S1: nsEM EM characterization of CODV_Ig_ and its complexes with IL4 and/or IL13. (a)** Representative nsEM micrograph of *apo*-CODV_Ig_ stained with 2% SST. Views of some individual antibodies are highlighted (scale bar: 500 Å). (**b**) representative 2D classes for CODV_Ig_ and its complexes with IL4 and/or IL13 reveals the absence of antigen density (scale bar: 100 Å). (**c**) Commensurate with the observations in **b**, the subsequent 3D reconstructions obtained also show no evidence of bound antigens (scale bar: 50 Å). The 3D reconstructions are fitted with the crystal structures of CODV_Fab_ (PDB: 5HCG) and IgG-Fc (PDB: 4NQS).

**
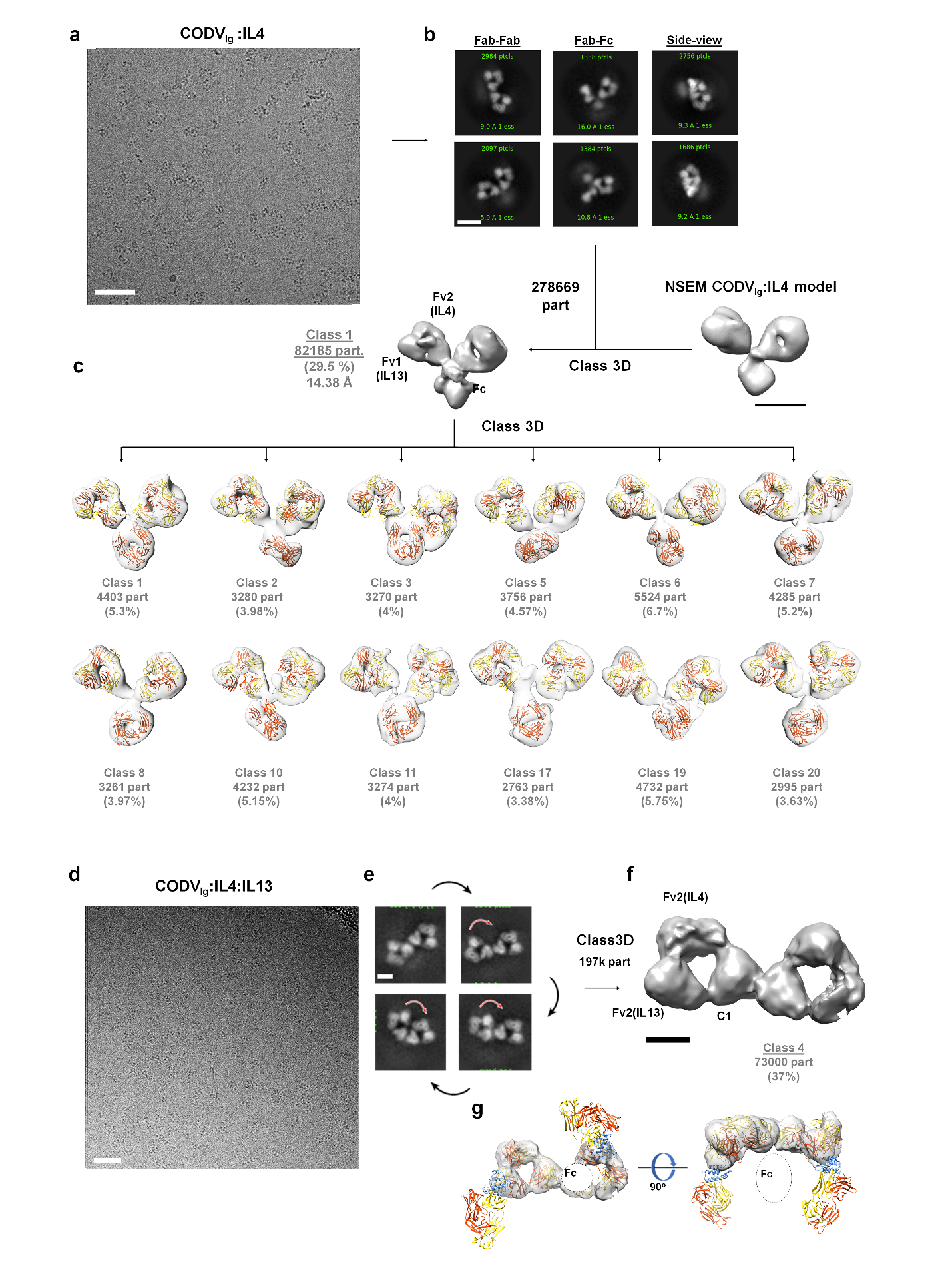
**

Fig. S2: Cryo-EM analysis of CODV_Ig_:IL4 and CODV_Ig_:IL4:IL13 complexes. (a) Representative micrograph (scale bar = 500 Å) and (b) 2D classes (bar = 150 Å) for CODV_Ig_:IL4. (c) 3D classification process revealed a high level of flexibility of the antibody, which precludes high resolution single particle reconstruction. (d) Representative micrograph of CODV_Ig_:IL4:IL13. (bar = 500 Å) (e) 2D classes CODV_Ig_:IL4:IL13 presented only one view, albeit with different orientations of the Fab regions (red arrows) along a 90^o^ motion (black arrows) (bar = 50 Å). (f) The lack of Fc density and different orientations in any of the classes led to reconstructions of only the two Fab regions. For both CODV_Ig_:IL4 and CODV_Ig_:IL4:IL13 no density was visible for the antigens.

**
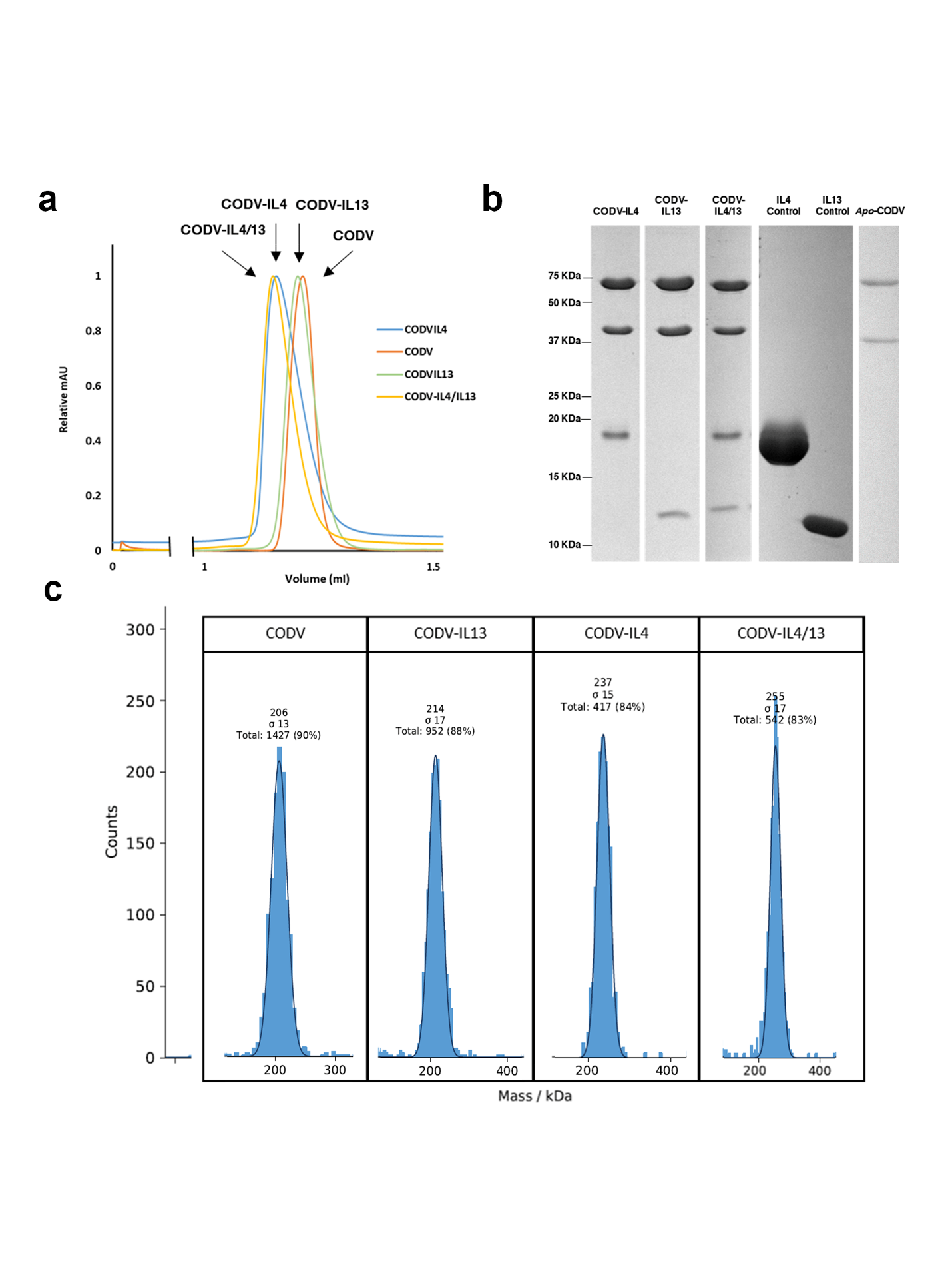
**

| Sample | CODV_Ig_ | CODV_Ig_:IL13 | | CODV_Ig_:IL4 | | CODV_Ig_:IL4:IL13 | | | |
| --- | --- | --- | --- | --- | --- | --- | --- | --- | --- |
| Expected Stoichiometry | - | 1:1 | 1:2 | 1:1 | 1:2 | 1:1:1 | 1:1:2 | 1:2:1 | 1:2:2 |
| Theoretical MW (kDa) | 198 | 210 | 222 | 214.5 | 231 | 226.5 | 238.5 | 243 | 255 |
| Experimental MW (kDa) | 206 | 214 | | 237 | | 255 | | | |
| Estimated Stoichiometry | - | 1:1 | | 1:2 | | 1:2:1 | | | |

**Fig. S3: Purification and characterization of CODV_Ig_ complexes**. **(a)** CODV_Ig_ and its complexes were purified by size-exclusion chromatography, with peaks relatively close to each other. (**b**) Each peak was confirmed for complex formation by SDS-PAGE. The uncropped gels corresponding to lanes 1-4 are in Fig. S8A and lane 5 is in Fig. S8B (**c**) Molecular weight determination of *apo*-CODV_Ig_ and complexes determined from *n* = 3 replicate mass photometry measurements. Shown below is a table of expected vs. average observed MW values for each antibody complex.


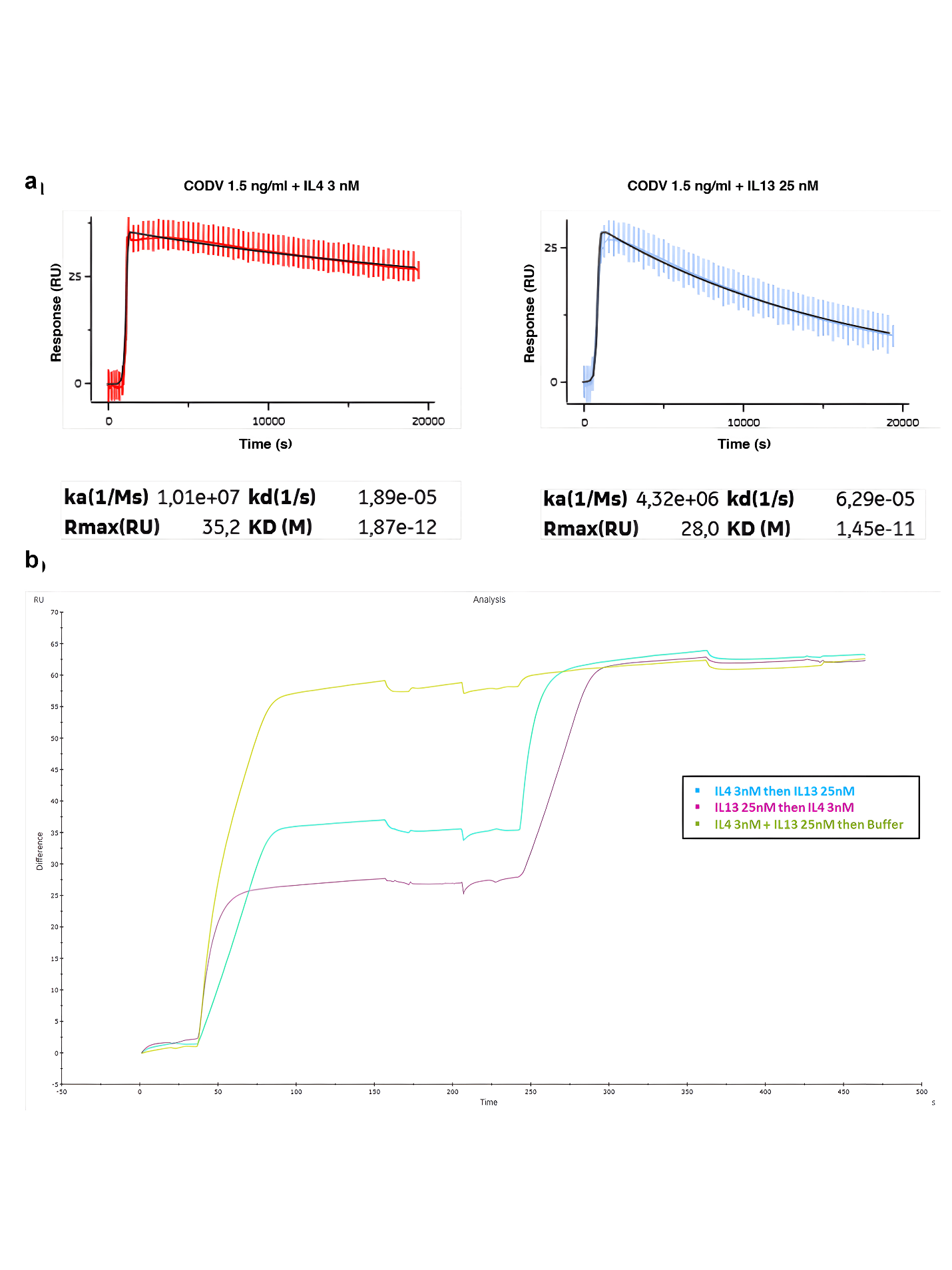


**Fig. S4: SPR evaluation of IL4 and IL13 binding to CODV_Ig_.** **(a)** IL4 (red) and IL13 (blue) were added in excess to CODV and after full binding, buffer was flowed for 5 hours. Binding constants (K_D_) show very high affinity for both interleukins, with a 10-fold lower affinity for IL13. (**b**) Binding of both antigens added in any order (blue, violet) or together (green) produces the same maximal response, suggesting no positional effect regarding antigen binding by CODV_Ig_.


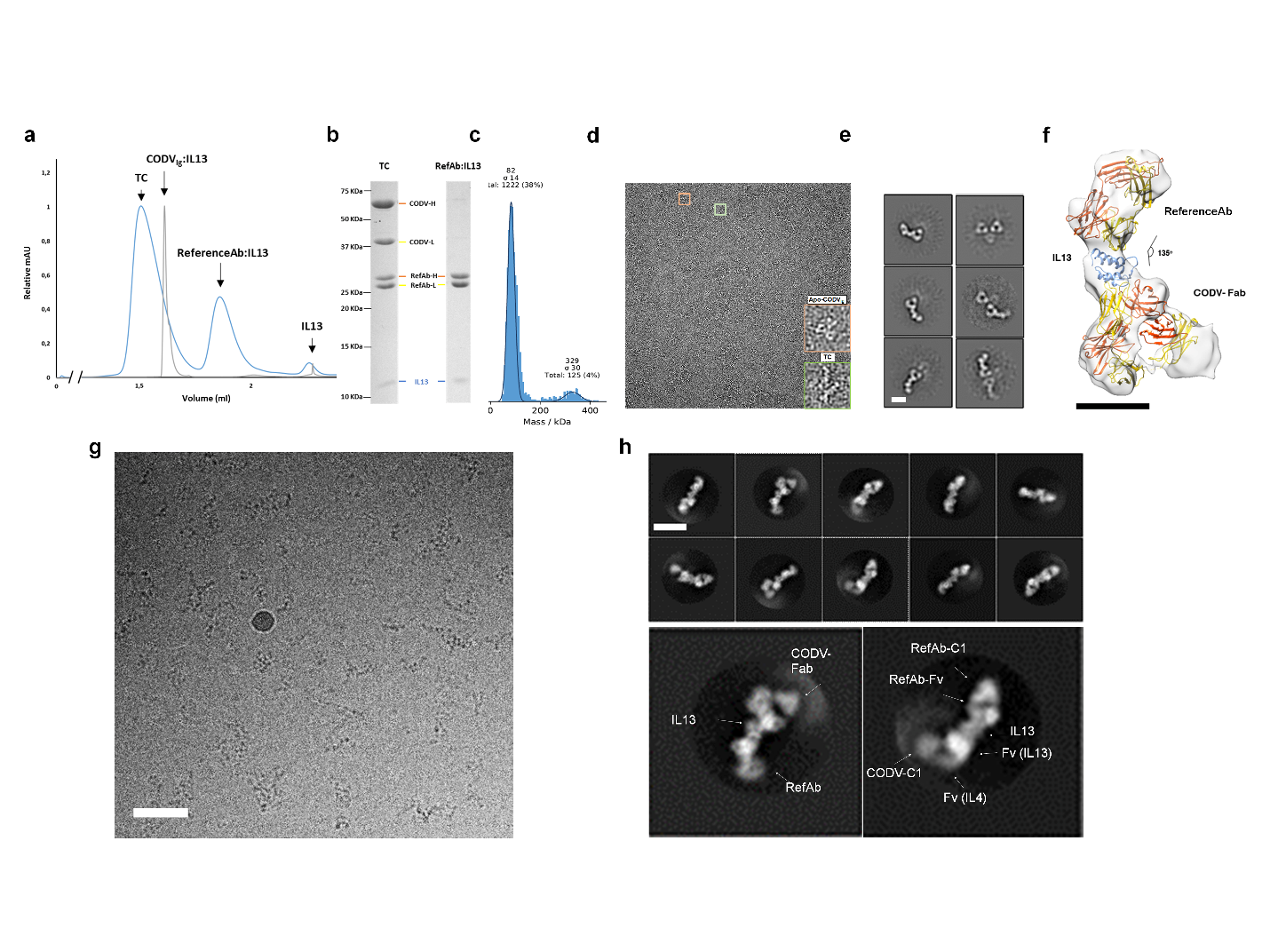


Fig. S5: (a) the SEC elution peak of CODV_Ig_:IL13:RefAb_Fab_ shows a clear shift compared to that observed for CODV_Ig_:IL13. (b) SDS-PAGE analysis of CODV_Ig_:IL13:RefAb_Fab_ and excess RefAb_Fab_:IL13 confirms ternary complex. The uncropped original gel picture is presented in Fig. S9 (c) Mass photometry analysis of TC (d) Representative nsEM micrograph of CODV_Ig_:IL13:RefAb_Fab_ contains images of *apo*-CODV and CODV_Ig_:IL13:RefAb_Fab_ with both 1:1:1 and 1:2:2 stoichiometries (scale bar: 500 Å). Insets: top: *apo*-CODV, bottom: TC with 1:1:1 stoichiometry. More examples of single molecule images showing a 1:1:1 stoichiometry and additional class averages are shown in Fig. S10 A and B, respectively. (e) the resulting 2D classes support this observation, but with a 1:1:1 stoichiometry dominating (scale bar: 100 Å). (f) Representative 3D class for CODV_Ig_:IL13:RefAb_Fab_ when using a Fab-focused approach fitted with crystal structures of CODVFab (PDB: 5HCG) and RefAb-IL13 (PDB: 5L6Y), which also shows densities of both antibodies and ligand (scale bar: 50 Å). (g) Top panel: Representative aligned averaged micrograph of 250 μg/ml TC (scale bar: 500 Å), (h) Gallery of aligned 2D class averages in different orientations (scale bar: 200 Å). Bottom panel: Illustration of all the individual domains, including IL13, of the TC that can be identified in the 2D class averages.


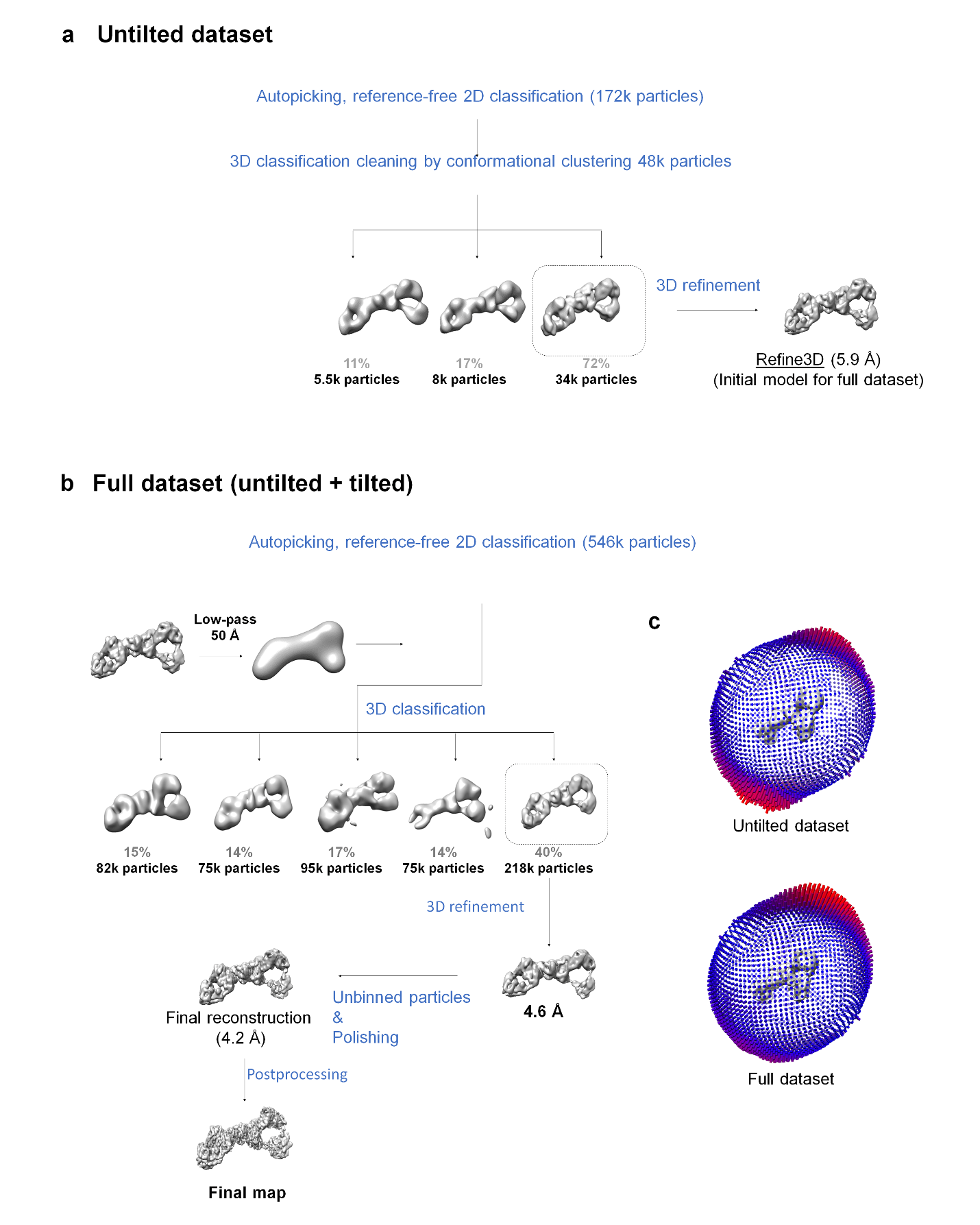


Fig. S6: Cryo-EM data processing workflow for CODV_Ig_:IL13:RefAb_Fab_. (Aa) A more strict cleaning by conformational isolation performed on the untilted particle set led to a 48k particle set. 3D refinement of the isolated class produced a reconstruction at 5.9A resolution, which was used as an initial model for an iterative classification of the tilted + untilted particle set. (b) 3D classification resulted in one class with higher percentage of particles which was further refined to a final map with a global resolution of 4.2 Å. (c) While still exhibiting preferential orientation, the full dataset improves the view completeness or population of particles at the originally less represented angles.


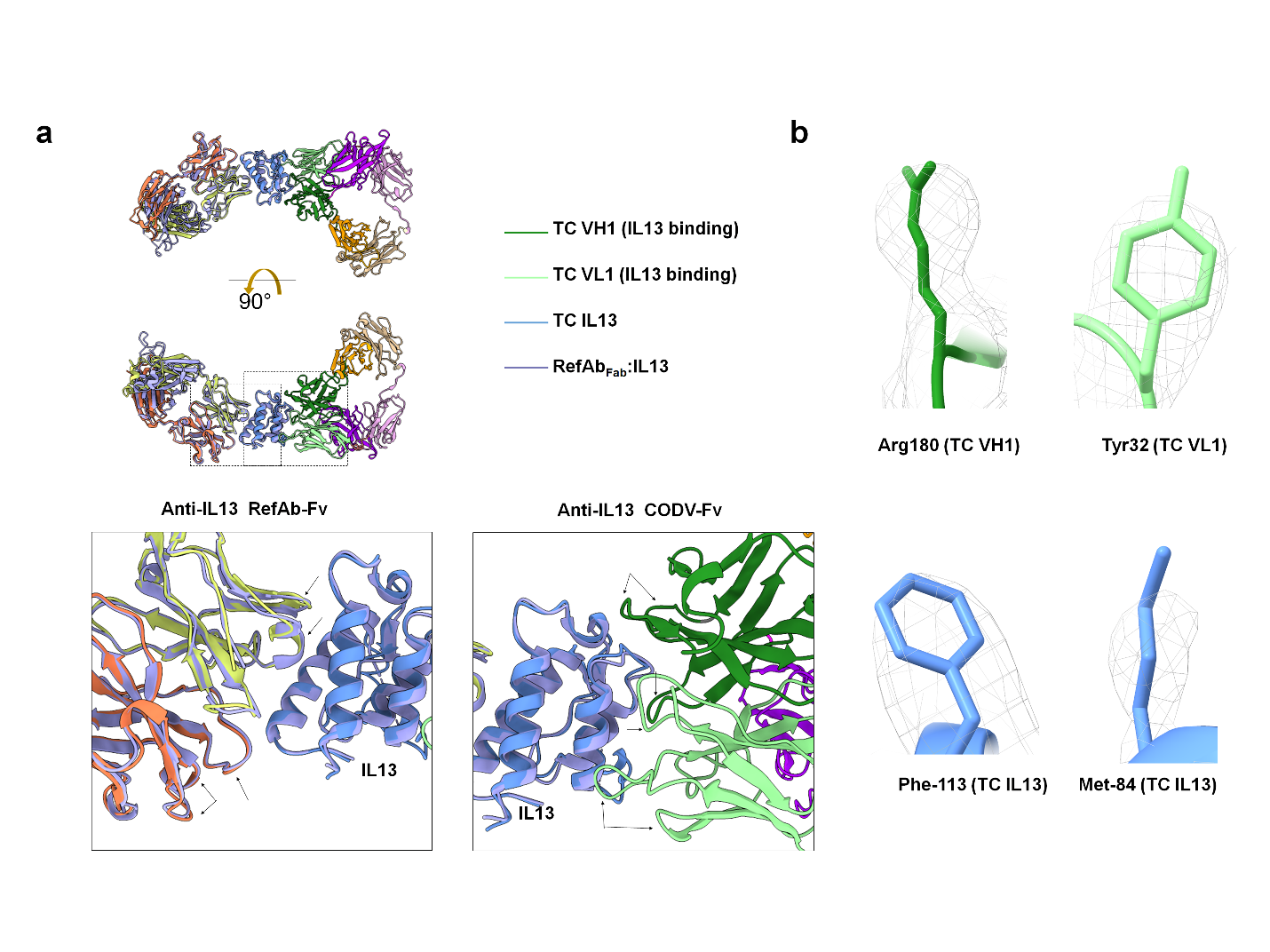


Fig. S7: Superposition of the current TC model and the crystal structure of RefAb:IL13^13^. (a) The antigen-antibody interfaces seem identical at the mainchain level. CDRs (black arrows) are conserved even when an additional Fab is bound. (b) Illustration of the map quality at the CODV_Fab_-IL13 interface.


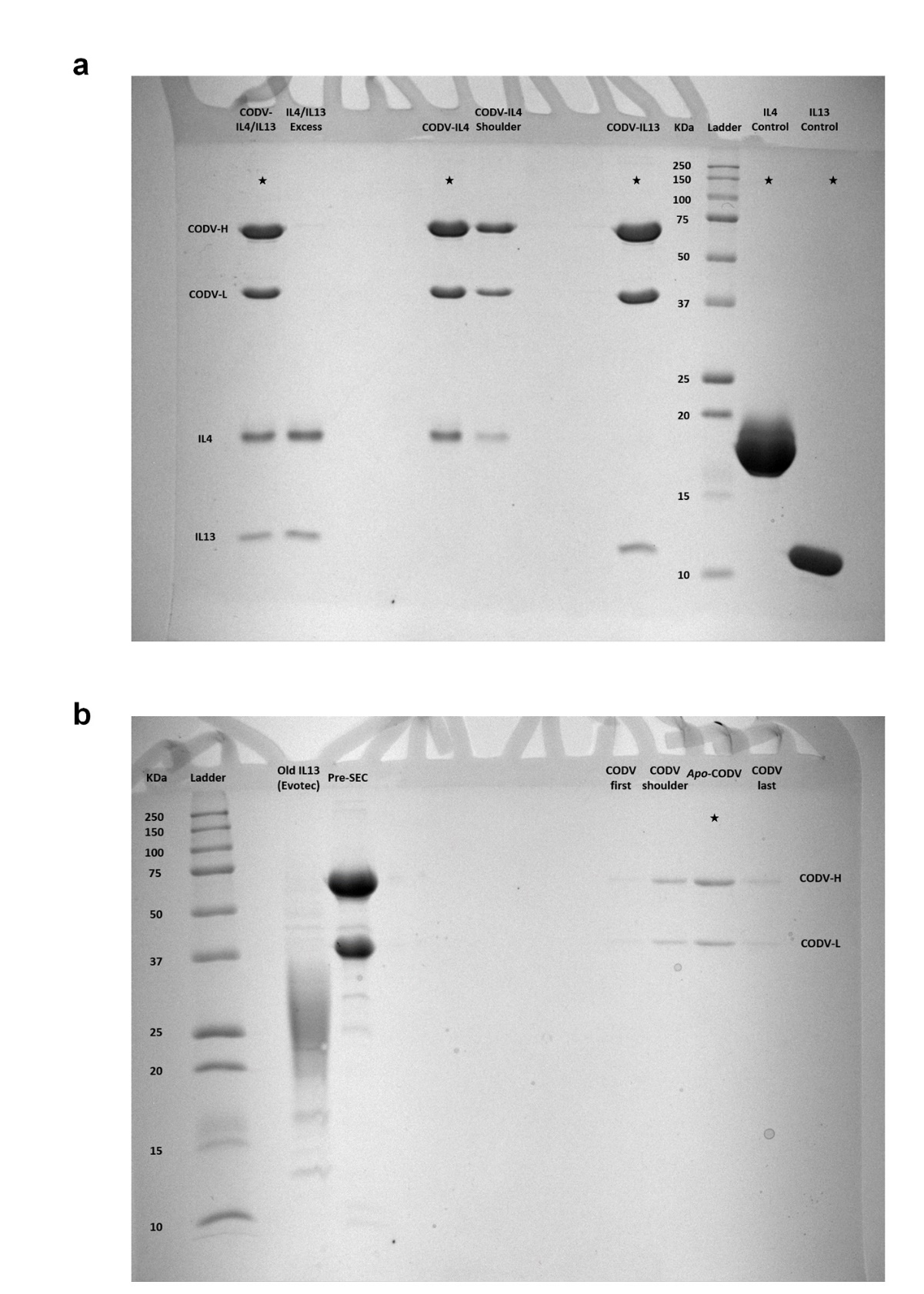


Fig. S8: Uncropped original SDS-PAGE picture related to Fig. S3. (a) This gel picture shows the complexes corresponding to the lanes 1-4 of the SDS-PAGE in Fig. S3B. The respective lanes are marked each by an asterisk (b) This gel contains the Apo-CODV (lane 5) of SDS-PAGE in Fig. S3B. The respective lane is marked by an asterisk.


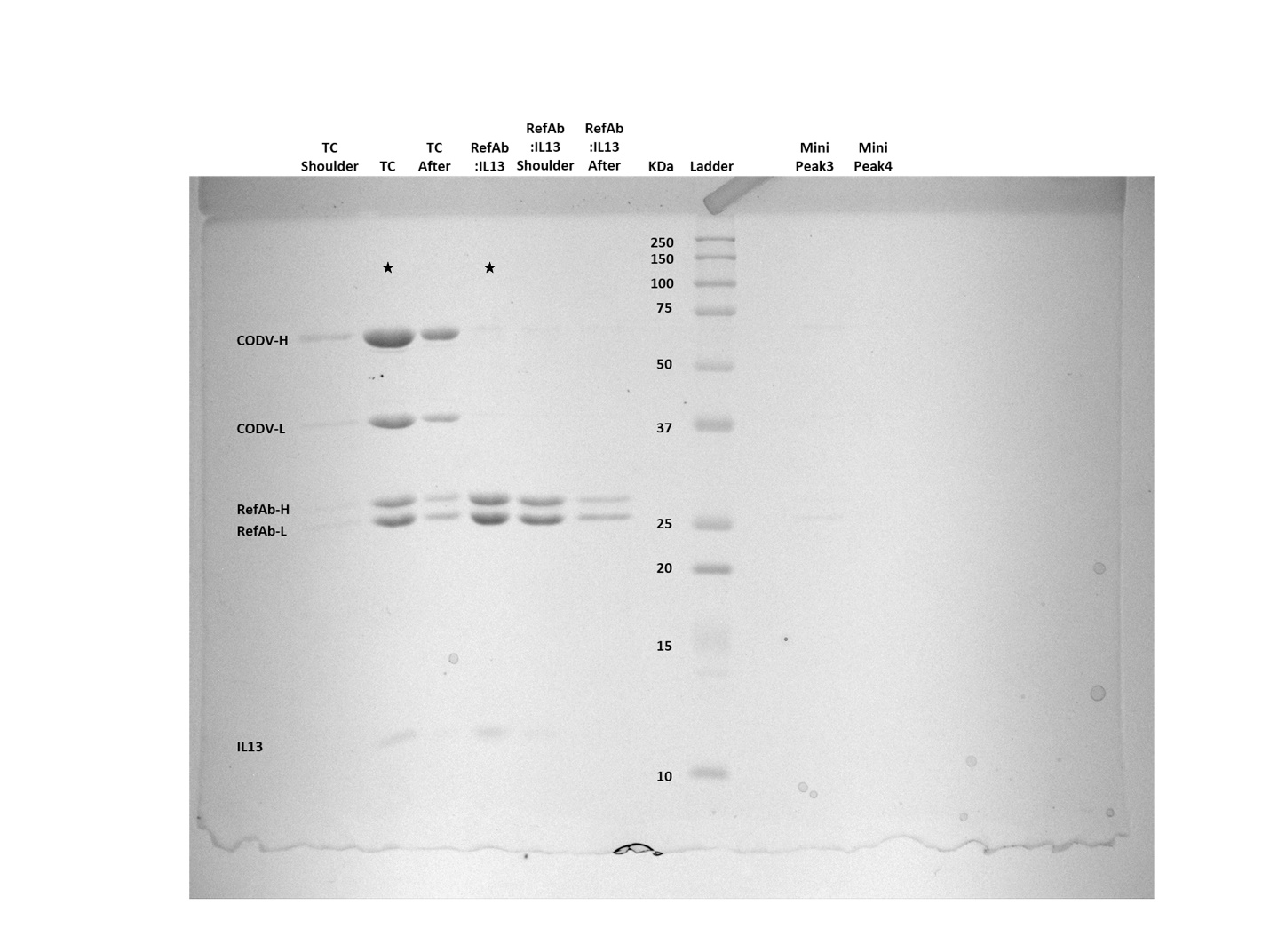


Fig. S9: Uncropped original SDS-PAGE picture related to Fig. S5A: The respective lanes of TC and the RefAb:IL13 in the SDS-PAGE of Fig. S5B. are marked by an asterisk.


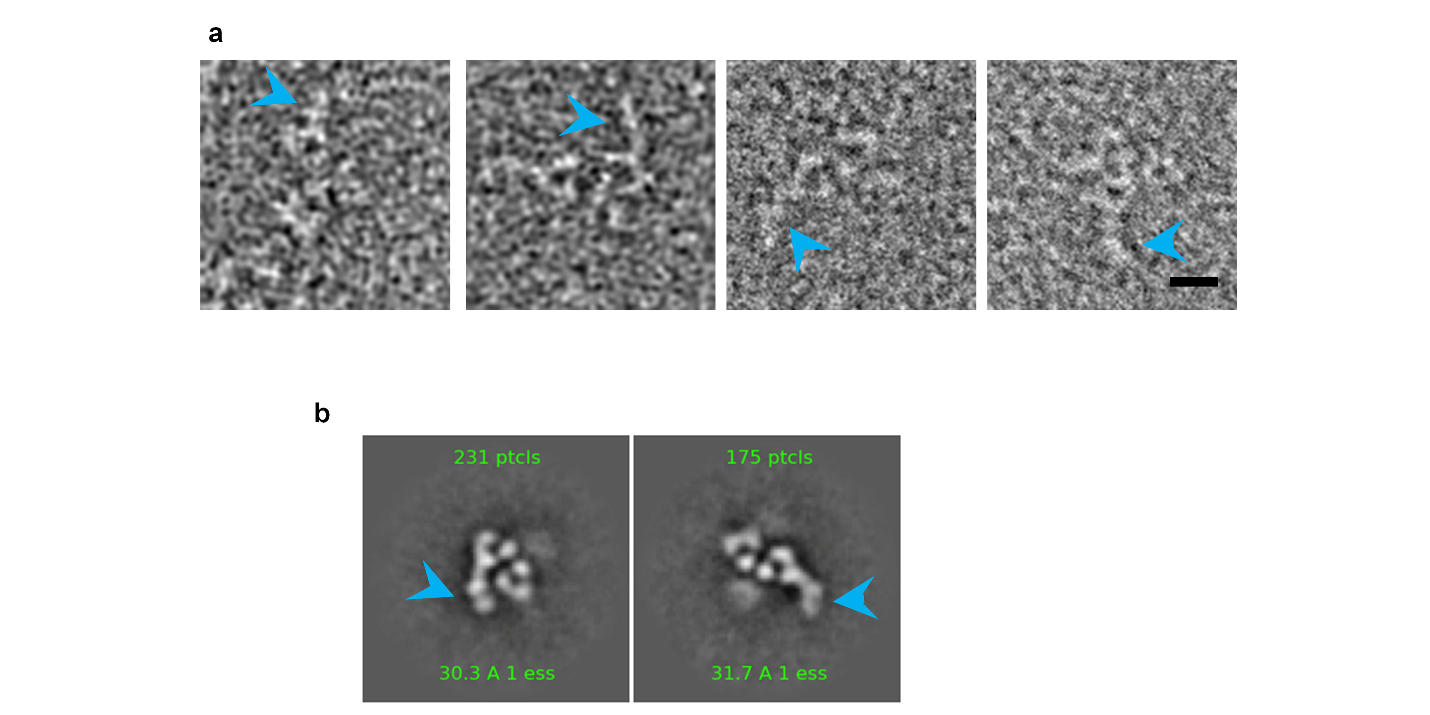


Fig. S10: Stoichiometry of the triple complex (TC): (a) NS-EM single molecule images showing 1:1:1 stoichiometry for TC, CODV_Ig_:IL13:ReferenceAb. Scale bar is 10nm (b) 2D class averages showing the 1:1:1 stoichiometry. ReferenceAb is marked by blue arrows in both A and B.

**SUPPLEMENTARY TABLES**

**Table S1:** Concentrations of IL4 and IL13 used against 1.5 µg/ml of CODV_Ig_ in each of the binding runs of SPR to determine the KD of ligand binding.

| **Dilution** | **1** | **2** | **3** | **4** | **5** | **6** | **7** | **8** |
| --- | --- | --- | --- | --- | --- | --- | --- | --- |
| **IL4 (nM)** | 3·10^-4^ | 0.001 | 0.006 | 0.032 | 0.16 | 0.8 | 4 | 20 |
| **IL13 (nM)** | 3·10^-3^ | 0.013 | 0.064 | 0.32 | 1.6 | 8 | 40 | 200 |

**Table S2:** Refinement statistics for the CODVF_rab_-IL13-RefAb (TC) cryo-EM dataset

| EM data collection and reconstruction | |
| --- | --- |
| Microscope | Titan Krios G3 |
| Voltage (kV) | 300 |
| Detector | K2 Summit |
| Magnification | 165,000 |
| Pixel size (Å/pix) | 0.83 |
| Frames per exposure | 40 |
| Exposure (e^-^/Å^2^) | 46 |
| Defocus range (μm) | -1.20 to -3.0 |
| Micrographs collected | 15198 |
| Particles extracted/used | 546717/218792 |
| Map sharpening B-factor (Å^2^) | -97.066 |
| Unmasked resolution at 0.5/0.143 FSC (Å) | 6.87/4.6 |
| Masked resolution at 0.5/0.143 FSC (Å) | 4.75/4.2 |
| Model refinement and validation |  |
| Composition |  |
| Amino acids | 1220 |
| Atoms | 9210 |
| RMSD bonds (Å) (# > 4σ) | 0.002 (0) |
| RMSD angles (^o^) (# > 4σ) | 0.550 (0) |
| Mean B-factors | 160.62 |
| Ramachandran |  |
| Outliers (%) | 0.08 |
| Allowed (%) | 4.88 |
| Favoured (%) | 95.04 |
| Rotamer outliers | 0.00 |
| Cβ outliers (%) | 0.00 |
| CaBLAM outliers (%) | 3.67 |
| MolProbity score | 1.93 |
| Clash score | 11.94 |
| CC (mask) | 0.82 |

**Table S3:** Residues of the CDR loops of VL1 and VH1 recognizing IL13 according to IMGT definition.

| **IL13 recognition** | **Heavy chain (Residue #)** | **Light chain (Residue #)** |
| --- | --- | --- |
| **CDR1** | GFSLTDSS (150-157) | ESVDSYGQSY (27-36) |
| **CDR2** | IWGDGR (175-180) | LAS (51-53) |
| **CDR3** | ARDGYFPYAMDF (220-231) | QQNAEDSRT (93-101) |

**Table S4:** SAXS Data table

| **Data-collection parameters** |  |
| --- | --- |
| Instrument | ESRF BM29 |
| Wavelength (Å) | 0.99 |
| q-range (Å^-1^) | 0.007-0.5 |
| Sample-to-detector distance (m) | 2.6 |
| Concentration range (mg/mL) | 1-6 |
| Temperature (K) | 298 |
| Detector | Pilatus 1M |
| Flux (photons/s) | 1.4*10^12^ |
| Beam size at sample (µm) | 700*700 |
|  |  |
| **Structural parameters (HPLC)** | **CODV_Ig_:IL13:RefAb_Fab_** |
| I0 (kDa) [from Guinier] | 45.6 |
| Rg (Å) [from Guinier] | 76.69 +/- 2.41 |
| Rg (Å) [from P(r)] | 79.29 |
| qminRg - qmaxRg used for Guinier | 1.2*10^-2^-1.6*10^-2^ |
| Volume (Å^3^) | 4.1*10^5^ |
| D_max_ (Å) | 306.5 |
| Calculated MW (kDa): | 220-270 |
|  |  |
| **Software employed** |  |
| Primary data reduction: | ScatterIV |
| Data processing | ScatterIV, ATSAS DAMMIF |
